# Flexible cue anchoring strategies enable stable head direction coding in both sighted and blind animals

**DOI:** 10.1038/s41467-022-33204-0

**Published:** 2022-09-19

**Authors:** Kadjita Asumbisa, Adrien Peyrache, Stuart Trenholm

**Affiliations:** Montreal Neurological Institute, McGill University, Montreal, QC H3A 2B4 Canada

**Keywords:** Visual system, Neural circuits, Olfactory system, Sensorimotor processing

## Abstract

Vision plays a crucial role in instructing the brain’s spatial navigation systems. However, little is known about how vision loss affects the neuronal encoding of spatial information. Here, recording from head direction (HD) cells in the anterior dorsal nucleus of the thalamus in mice, we find stable and robust HD tuning in rd1 mice, a model of photoreceptor degeneration, that go blind by approximately one month of age. In contrast, placing sighted animals in darkness significantly impairs HD cell tuning. We find that blind mice use olfactory cues to maintain stable HD tuning and that prior visual experience leads to refined HD cell tuning in blind rd1 adult mice compared to congenitally blind animals. Finally, in the absence of both visual and olfactory cues, the HD attractor network remains intact but the preferred firing direction of HD cells drifts over time. These findings demonstrate flexibility in how the brain uses diverse sensory information to generate a stable directional representation of space.

## Abstract (web summary)

Vision plays an important role in the head direction cell system in animals. Here the authors recorded from head direction cells in rd1 mice that show retinal degeneration at 1 month, and find that they use smell cues to maintain stable HD tuning.

## Introduction

Our visual system provides critical and up-to-date information about the world around us, facilitating navigation through the environment and enabling quick reactions to dynamic events. Vision facilitates navigation-related tasks, including landmarking, obstacle avoidance, and the generation of an internal cognitive spatial map^1–4^. In the absence of vision, other sensory modalities need to fill the previously dominant role of vision in generating spatial awareness and guiding navigation. In humans, while it is clear that visually impaired individuals can successfully form spatial maps and navigate in many environments, behavioral studies have noted differences in various aspects of spatial awareness and navigation between sighted and visually impaired individuals^4–6^. However, little is known about how the brain’s spatial navigation systems, which have been best examined in freely moving rodent studies^7^, adapt following vision loss.

The brain has dedicated systems for generating spatial awareness and guiding spatial navigation. In rodents, some of the most studied components of the brain’s spatial awareness system include place cells^2^, grid cells^8^, and head direction cells^9^—although other complementary spatially-tuned cells also exist^7^. To examine the effect of vision loss on part of the brain’s spatial navigation system, we decided to focus on head direction (HD) cells in the anterior dorsal nucleus (ADn) of the thalamus, where the spike rate of a majority of neurons is modulated by head direction^10,11^, but the stability of the system following vision loss has not been explored.

The head direction system encompasses a network of interconnected brain regions that incorporate angular head velocity signals with additional sensory information about environmental landmarks in order to generate cells with highly tuned and stable preferences for head direction^12^. Ablation/silencing studies have shown that intact HD cells are required for proper spatial navigation^13–15^ and accurate spatial representation within the brain’s other spatial navigation systems^16,17^. The HD system appears to be organized as an attractor network^11,18–20^, such that pairs of simultaneously recorded HD cells maintain a similar angular difference between their preferred firing directions across exposures to different rooms and following environmental manipulations that alter tuning preferences^21^. As such, cells with similar HD tuning preferences tend to fire coherently even when tuning preferences are unstable^22^ or during sleep^11,19^.

Although vestibular inputs are critical for the HD signal^23–25^, vision also appears to play an important role in anchoring and providing stability to the HD system. For example, horizontally displacing a visual cue in an environment reliably causes a concomitant shift in the preferred firing direction (PFD) of HD cells^21^. However, many studies have reported that HD cell responses are relatively stable in the absence of visual inputs or visual cues^14,26–28^. Furthermore, recordings from HD cells in young rodents just before eye-opening revealed tuned HD cells, although tuning curves were found to be broader than following eye-opening^22,29,30^. It has thus been argued that the HD system only requires idiothetic inputs (i.e., internally generated sensory signals, such as vestibular, proprioceptive, or motor efference copy signals), with vision only providing a refining allothetic (i.e., externally generated) sensory input^12,31,32^. In contrast, other studies have reported highly unstable HD cell responses following the removal of visual inputs in adult rodents^33,34^, arguing for the possible requirement of external sensory inputs in stabilizing HD tuning. Additionally, while it is clear that vision can exert a strong effect on HD cell tuning preferences, the extent to which other sensory systems can provide landmarking cues and stabilize HD cell tuning remains unclear. While rodents have been found capable of using both auditory and olfactory cues to guide spatial learning and navigation^35–37^, previous studies with HD cells have suggested that auditory, olfactory, and vibrissal systems are relatively ineffective in modulating HD cell tuning^31,38^. Thus, it remains uncertain what effect vision loss might have on HD cell responses. Here, to examine the effect of vision loss on HD cells in adult mice, we recorded HD cells in ADn of both sighted and blind animals (including blind adult animals both with and without prior visual experience) and explored the extent to which HD cell responses were altered following vision loss, and whether other sensory systems could be leveraged to tune the HD system.

## Results

### Robust head direction tuning in blind mice

**Fig. 1:**
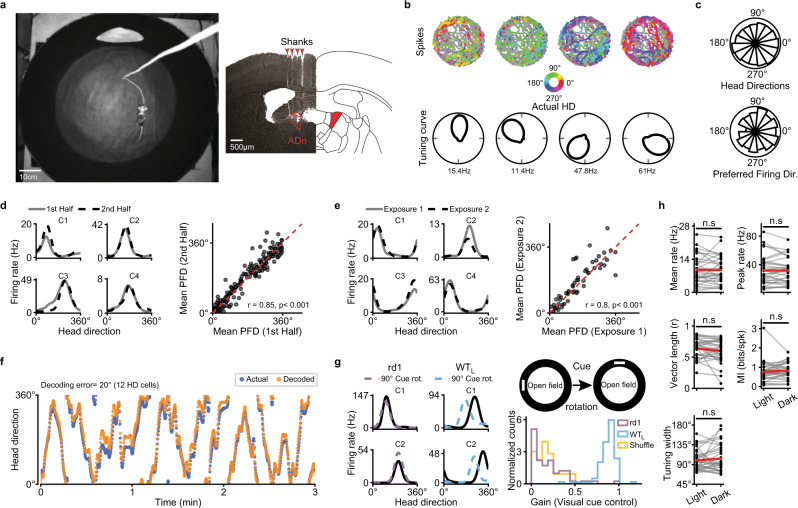
Robust and stable head direction cells in ADn of blind mice. **a** left, The open-field recording arena featuring a mouse with an electrode implant. **a** right, Example post hoc coronal brain slice showing tracts of the 4-shank recording electrode (left) with the anterior dorsal nucleus (ADn) indicated, and a corresponding slice from a brain atlas (right). **b** top, For 4 simultaneously recorded HD cells from an rd1 mouse, the animal’s position in the arena is plotted over a 10 min session (gray lines), and the spatial locations that evoked spiking responses are color-coded based on the animal’s head direction. **b** bottom, Polar plots indicating the preferred firing directions (PFDs) for the cells shown above. **c** top, Polar plot showing the occupancy of angular bins for head direction across 13 rd1 mice during 10 min recording sessions (see Methods). **c** bottom, Polar plot showing PFDs for HD cells recorded across 13 rd1 mice (see Methods). **d** left, Spike rate vs. head direction of four simultaneously recorded HD cells (C1–C4) in an rd1 mouse during the first/second half of a 10 min session. **d** right, For all cells/animals (*n* = 151 HD cells across 13 animals), the mean PFD is compared between the first/last 5 min. Pre-post tuning similarity was tested with a circular correlation. **e** The same as in **d**, except comparing HD cell tuning across successive 10 min exposures to the same arena (*n* = 55 HD cells across 7 animals). **f** Example decoding, comparing the actual head direction of an rd1 mouse over time (blue) to the head direction predicted by a Bayesian decoder (orange, see Methods). **g** Spike rate vs. head direction of 2 simultaneously recorded HD cells in an rd1 (left) and sighted (middle) mouse in control (solid black line) and following a 90° visual cue rotation (dotted line). **g** right, Schematic outlining the visual cue rotation experiment, and a histogram showing the extent of control (gain) that visual cue rotation exerted on the PFD of HD cells in rd1 vs. sighted mice (see Methods). Gain distributions for WT_L_ (C1: Gain = 0.81; C2: Gain = 0.75) and rd1 (C1: Gain = 0.07; C2: Gain = 0.01) mice were compared to a shuffled distribution (see Methods). rd1 mice were not statistically different than shuffled data (*P* = 0.28; two-sided Mann–Whitney U Test). WT_L_ were statistically different than shuffled data (*P* < 10^−57^; two-sided Mann–Whitney U Test). WT_L_ = 93 HD cells across 6 animals; rd1 = 65 HD cells across 6 animals. **h** Comparison of several HD cell metrics in rd1 mice during light vs. dark exposure (*n* = 31 HD cells across 4 animals). Statistical differences calculated using the two-sided Wilcoxon Signed-Rank Test. n.s not statistically different. Source data are provided as a Source Data file.

Is HD cell tuning stable following vision loss? To examine this, we recorded from neurons in ADn of rd1 mice^39^, a rodent model of retinitis pigmentosa in which mice are born with normal vision but go blind by ~1 month old, due to photoreceptor degeneration^40^. For all experiments, recording probes were implanted in ADn of adult mice (2–4 months old), and animals were placed in a circular open-field arena (Fig. 1a; 60 cm diameter; black walls with a single visual cue; see Methods). The animal’s position and head direction were tracked with a set of IR video cameras while spiking responses of HD cells were recorded (see Methods). Following experiments, spike sorting was performed to isolate individual HD cells, based on methods previously described for identifying HD cells in sighted animals (see Methods). Unless otherwise noted, HD cell responses were analyzed over the entirety of 10 min-long recording sessions (see Methods).

Upon placing blind adult rd1 mice in the open-field environment, we found robust HD cell tuning (Fig. 1b; *n* = 151 HD cells recorded from 13 animals, with 80.7% of recorded cells in ADn defined as HD cells; see Methods). Similar to what has been described for sighted animals, we found that rd1 mice evenly sampled all angular directions in the environment and possessed HD cells that exhibited preferred firing directions that spanned all head directions (Fig. 1c).

We next tested the stability of HD cells in blind mice and the extent to which their HD cell network was able to reliably encode head direction. First, we found that the preferred direction of HD cells remained stable over a single 10 min recording session (Fig. 1d), and across repeated exposure to the same arena (Fig. 1e), with levels of stability similar to what we found for sighted animals in the light (Supplementary Fig. 1). Next, we examined whether the HD cell network in ADn of blind mice was providing a reliable readout of the animal’s head direction by performing a decoding analysis^41,42^. We found that simultaneously recorded cells provided robust and stable decoding of HD (Fig. 1f). Therefore, in blind rd1 mice, HD cells are highly tuned and provide an accurate readout of head direction.

To ensure that rd1 animals were indeed blind, we performed visual cue rotation experiments in the light. Upon rotating a visual cue, HD cells in rd1 mice failed to follow the cue, unlike HD cells from sighted animals in the light (Fig. 1g). Furthermore, various metrics that we computed for HD cells in rd1 mice (e.g., mean and peak firing rates, resultant vector length, mutual information (MI), and tuning width) were statistically similar in both light and dark environments (Fig. 1h). Thus, despite a total absence of rod and cone based visual inputs, HD cell tuning is robust and stable in blind rd1 animals, providing accurate information about head direction, meaning that blind mice can generate a stable allocentric spatially guided map.

### HD cell tuning is more robust in blind animals than in sighted animals placed in the dark

**Fig. 2:**
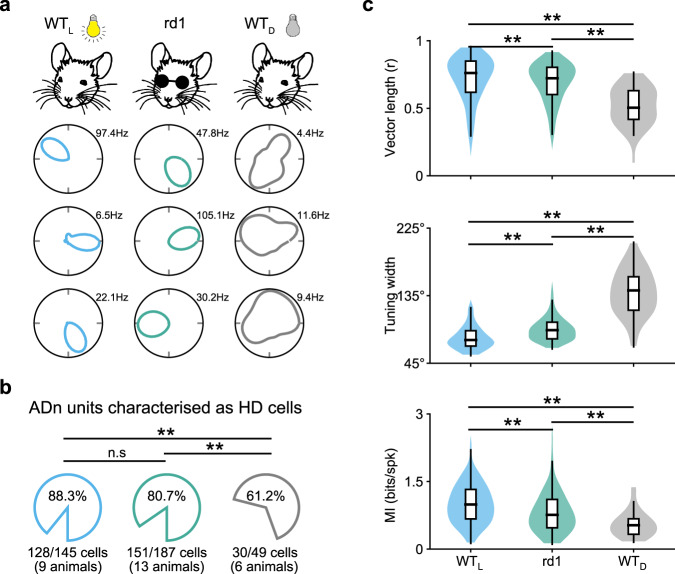
HD cell tuning is more robust in blind animals than in sighted animals placed in the dark. **a** For three different groups of mice—wild-type in light (WT_L_; blue), rd1 (blind; green), and wild-type in the dark (WT_D_; gray)—polar plots of four simultaneously recorded HD cells are shown. **b** For the three different groups of mice outlined in **a**, the average percent of cells recorded in ADn that passed the criterion to be designated as HD cells (see Methods) are compared. Statistical differences were calculated using a two-sample Z-test for Proportions: WT_L_ vs rd1, *P* = 0.06; WT_L_ vs WT_D_, *P* < 10^−4^; rd1 vs WT_D_, *P* = 0.004. **c** For the three different groups of mice outlined in **a** and **b**, several characteristics of HD cells are compared. For each graph, data are shown as hybrid violin/box plots (see Methods). Statistical differences were calculated using the two-sided Mann–Whitney U Test with Bonferroni correction for multiple comparisons: Vector length (WT_L_ vs rd1, *P* < 10^−2^; WT_L_ vs WT_D_, *P* < 10^−7^; rd1 vs WT_D_, *P* < 10^−4^); Tuning width (WT_L_ vs rd1, *P* < 10^−9^; WT_L_ vs WT_D_, *P* < 10^−12^; rd1 vs WT_D_, *P* < 10^−11^); MI (WT_L_ vs rd1, *P* < 10^−3^; WT_L_ vs WT_D_, *P* < 10^−6^; rd1 vs WT_D_, *P* = 0.002). The number of cells and animals in each group is outlined in panel **b**. Additional statistical comparisons are provided in Supplementary Fig. 2. Source data are provided as a Source Data file.

How do HD cell responses in blind animals compare to those of sighted animals? To test this, we recorded HD cell responses from sighted animals in the light (WT_L_) and in complete darkness (WT_D_; see Methods; Fig. 2a). A similar percentage of cells recorded in ADn passed the criteria to be designated as HD cells in rd1 and WT_L_ mice (Fig. 2b; see Methods), whereas a significantly smaller percentage of ADn cells were designated as HD cells in WT_D_ mice (Fig. 2b). Thus, HD cell tuning is significantly more robust in blind animals than sighted animals placed in the dark, meaning that in our experimental arena blind animals have adapted an alternative non-visual strategy for stabilizing HD cell tuning.

Next, we compared several metrics for HD cell responses between blind and sighted mice. For vector length, tuning width, and mutual information, each group of mice was statistically different from one another, demonstrating the following hierarchy in HD tuning refinement: WT_L_ > rd1 > WT_D_ (Fig. 2c; Supplementary Fig. 2a, b). Furthermore, if we limited our analysis in WT animals to HD cells that were recorded in both light and dark environments, and performed paired statistical tests, we found a similar relationship with HD tuning being significantly impaired for WT animals in the dark compared to the light (Supplementary Fig. 2c). Thus, sighted mice exhibit significant impairment in HD cell tuning when placed in the dark, with a level of impairment much more severe than is seen in blind animals, for whom HD cell tuning—although slightly less refined than in sighted animals in the light—is robust and stable.

### Prior visual experience leads to refined HD cell tuning in blind adult mice

**Fig. 3:**
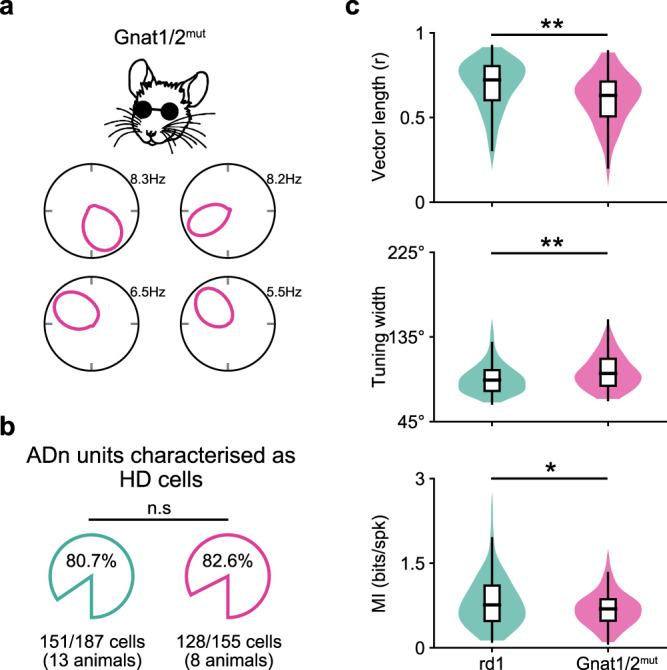
Prior visual experience leads to refinement of HD cell tuning in blind adult mice. **a** For a Gnat1/2^mut^ mouse (congenitally blind; pink), polar plots of four simultaneously recorded HD cells are shown. **b** For rd1 (replotted from Fig. 2) and Gnat1/2^mut^ mice, the average percent of cells recorded in ADn that passed the criterion to be designated as HD cells (see Methods) are compared. Statistical differences calculated using the two-sample Z-test for Proportions. **c** Several characteristics of HD cells are compared between rd1 (replotted from Fig. 2) and Gnat1/2^mut^ mice. For each graph, data are shown as hybrid violin/box plots (see Methods). Statistical differences were calculated using the two-sided Mann–Whitney U Test (Vector length, *P* < 10^−5^; Tuning width, *P* = 0.0005; MI, *P* = 0.014), and the number of cells and animals in each group is outlined in panel **b**. Additional statistical comparisons are provided in Supplementary Fig. 3. Source data are provided as a Source Data file.

We next tested whether vision might be required during development for the proper maturation of HD cell tuning, even if mice subsequently go blind. For instance, in the superior colliculus, it has been shown that normal vision is required during development for the proper formation of auditory maps^43,44^. In mice, eye-opening occurs ~P10–12^45,46^, and rd1 mice have attenuated vision for a few days following eye-opening until they go fully blind around 1 month old^40,47^. To test whether vision around the time of eye-opening is required to refine and mature the tuning of the HD system, we performed experiments in Gnat2^cpfl3^ Gnat1^irdr^/Boc mice (subsequently referred to as Gnat1/2^mut^), who are congenitally blind due to dysfunctional rod and cone photoreceptors (see Methods). Recording from adult Gnat1/2^mut^ mice (2–4 months old), we found many highly tuned HD cells (Fig. 3a; *n* = 128 HD cells recorded from 8 animals) with a similar percentage of cells in ADn designated as HD cells for Gnat1/2^mut^ mice as we found for rd1 mice (Fig. 3b). Similar to rd1 mice, HD cells in Gnat1/2^mut^ mice maintained stable preferred firing directions throughout a 10 min session, as well as across repeated exposure to the same environment (Supplementary Fig. 3a). Furthermore, similar to rd1 mice, responses and tuning of HD cells in Gnat1/2^mut^ mice were statistically similar in both light and dark conditions (Supplementary Fig. 3b). Finally, at the population level, simultaneously recorded HD cells provided a reliable decoding of the animal’s head direction (Supplementary Fig. 3c). However, based on the metrics that we calculated from HD cell responses during exploration of the open-field environment, we found that HD cell tuning was statistically less refined in Gnat1/2^mut^ mice compared to rd1 mice on several metrics tested (Fig. 3c; Supplementary Fig. 3d,e). These results indicate that while visual inputs are not required for generating stable HD tuning in adult blind mice—either in rd1 or Gnat1/2^mut^ animals—normal visual inputs in the days after eye-opening appear to be required for refinement and maturation of vision-independent HD tuning in adult blind mice.

### The vibrissal system is not required for head direction cell tuning in blind animals in the open-field environment

**Fig. 4:**
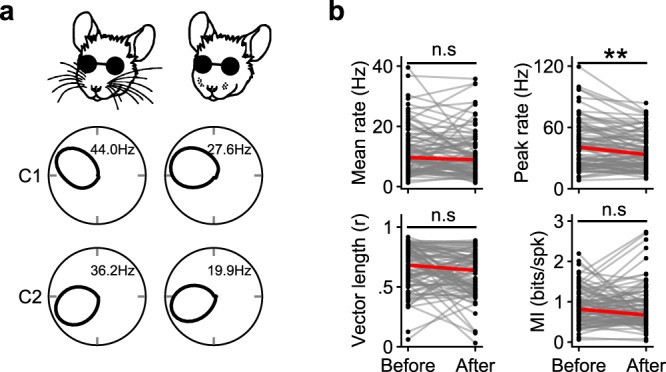
Stable HD cell tuning in blind animals following whisker shaving. **a** Example polar plots of two simultaneously recorded HD cells in an rd1 mouse before (left) and after (right) whisker ablation. **b** Several HD cell metrics are compared before and after whisker ablation, pooled for rd1 and Gnat1/2^mut^ (independent analyses for these different mouse lines are shown in Supplementary Fig. 4). Statistical comparisons were computed using the two-sided Wilcoxon Signed-Rank Test: *N* = 89 HD cells across 11 animals (Mean rate, *P* = 0.07; Peak rate, *P* = 0.0001; Vector length, *P* = 0.14; MI, *P* = 0.68). Source data are provided as a Source Data file.

We next tested whether blind mice were using an alternative sensory modality to landmark their HD system within the open-field environment. As all our experiments were performed with a speaker playing white noise placed immediately underneath the center of the open-field arena, we deemed it unlikely that auditory inputs were contributing to HD cell tuning in our experiments (see also ref. 31). We first examined the vibrissal system, hypothesizing that a mouse might be surveying the walls and floor of the open-field arena with its whiskers to aid in spatial awareness. To test this, we shaved the whiskers from blind mice (see Methods) and examined the effect on HD cell responses (Fig. 4a). Whisker shaving did not result in any noticeable change in HD cell tuning, except for a reduction in the peak firing rate (Fig. 4a, b; we pooled together rd1 and Gnat1/2^mut^ mice as HD cells in both were similarly unaffected by whisker shaving; Supplementary Fig. 4a, b). Whisker shaving similarly had no effect on HD cell tuning of sighted animals in the light (although, again, it led to a small reduction in peak firing rate; Supplementary Fig. 4c). Thus, in our experimental arena, the vibrissal system does not appear to play a major role in HD cell tuning in either blind or sighted animals.

### The olfactory system is required for stabilizing head direction cell tuning in blind animals

**Fig. 5:**
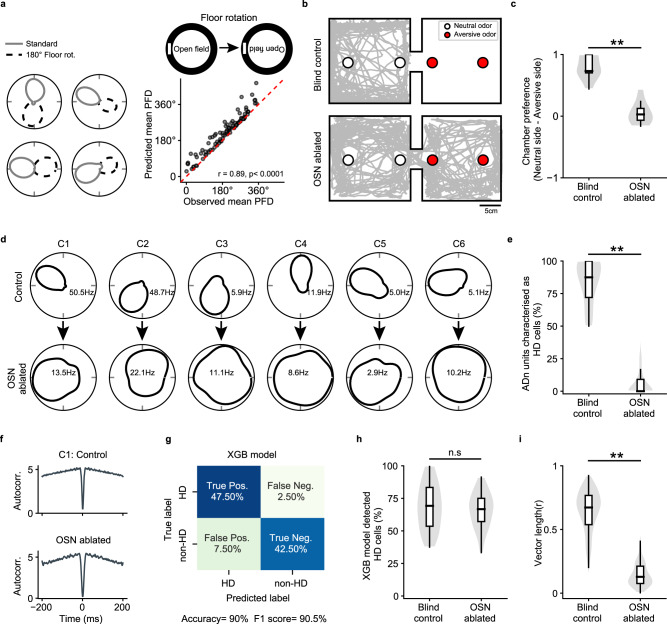
Olfactory signals are required for stable HD cell tuning in blind animals. **a** left, Polar plots from four simultaneously recorded HD cells in an rd1 mouse before (solid gray line) and after (dotted black line) 180° floor rotation (see Methods). **a** right (top), Schematic of floor rotation experiment. **a** right (bottom), Observed shift in mean PFD plotted against expected shift in mean PFD (see Methods; pooled for rd1 and Gnat1/2^mut^ (see also Supplementary Fig. 5a, b)). r-value computed with circular correlation (94 HD cells across 9 animals). **b** Example olfactory test, with an rd1 mouse placed in a two-room chamber, with one room containing an aversive odor (3-methyl-1-butanethiol) and the other containing a neutral odor (distilled H_2_0). The mouse’s trajectory is plotted (gray line) over a 10 min session in control (left) and following olfactory sensory neuron (OSN) ablation (right). **c** Time spent in the neutral vs. aversive room in control and following olfactory ablation. Statistical test used was the two-sided Mann–Whitney U Test, *P* = 0.0006. Blind control = 8 animals; Olfaction ablated = 9 animals. **d** Polar plots for six simultaneously recorded HD cells in an rd1 mouse in control (top) and the following day after OSN ablation (bottom; see Methods). **e** Violin/box plots comparing the percent of cells in ADn passing standard criterion for being designated HD cells (see Methods) in control and following olfactory ablation. Statistical test used was the two-sided Mann–Whitney U Test, *P* < 10^−12^. Blind control = 21 animals; Olfaction ablated = 9 animals. **f** Example autocorrelograms for an HD cell (C1) in a blind animal in control (top) and following olfactory ablation (bottom). **g** Results from the XGB model, trained on autocorrelograms of sighted animals in the light (WT_L_) and used to classify cells in ADn of blind animals as either HD and non-HD cells (see Methods). **h** Violin/box plots comparing the percent of ADn cells classified as HD cells using the XGB classifier in control and following olfactory ablation (see Methods; Blind control = 21 animals; Olfaction ablated = 9 animals). Two-sided Mann–Whitney U Test, *P* = 0.57. **i** Violin/box plots comparing the vector length calculated from XGB-classified HD cells in blind control vs. olfaction ablated animals. Blind control = 254 HD cells across 21 animals; Olfaction ablated = 179 HD cells across 9 animals. Statistical tests performed using the two-sided Mann–Whitney U Test, *P* < 10^−64^. Source data are provided as a Source Data file.

Due to the relative stability of preferred firing directions observed across repeated exposures that we had previously noted for blind mice, we suspected they might be using olfaction to anchor the HD system since the floor was not cleaned between repeated exposures. To test this, we performed a floor rotation experiment. Here, instead of rotating a visual cue on the wall as we did previously for visual cue rotation experiments, we rotated the floor (i.e., the animal was removed from the room, the floor was rotated without being cleaned, and the animal was reintroduced to the room). In blind mice, floor rotation resulted in a concomitant shift in the preferred firing direction of HD cells (Fig. 5a; we pooled together rd1 and Gnat1/2^mut^ mice as results were similar between these two blindness models; Supplementary Fig. 5a, b), suggesting that olfaction could modulate HD cell tuning. In blind animals, floor rotation led to concomitant shifts in the preferred direction of HD cells at levels similar to that observed in sighted animals in light following visual cue rotations (Supplementary Fig. 5).

To directly test whether olfactory inputs were required for stabilizing HD cell tuning in blind mice, we ablated olfactory sensory neurons (OSNs) and examined the effect on HD cell tuning. To ablate OSNs, we used a previously established chemical lesioning method^48^ (see Methods). To ensure that this method was effective at ablating OSNs and severely impairing olfaction, we developed an olfactory place avoidance task in which a mouse was placed in a two-chamber arena, with one of the chambers housing an aversive olfactory substance (3-methyl-1-butanethiol^49^) and the other chamber housing a neutral olfactory substance (distilled H_2_O; Fig. 5b; see Methods). Mice with intact OSNs completely avoided the chamber containing the aversive olfactory substance (Fig. 5b, c). In contrast, following OSN ablation, mice showed no preference between the two chambers (Fig. 5b, c). Having established a method to reliably ablate olfaction, we tested the effect of olfactory ablation on HD cell tuning in blind animals. Olfactory ablation resulted in complete loss of HD cell tuning in ADn of blind mice (Fig. 5d, e; we pooled together rd1 and Gnat1/2^mut^ mice as OSN ablation affected both similarly (Supplementary Fig. 5c, d)). The percent of ADn cells that passed the criteria for being designated as HD cells drastically decreased for blind mice following olfactory ablation (Fig. 5e, Supplementary Fig. 5e, f). For some experiments, whiskers were intact during olfactory ablation, while in other animals, whiskers were ablated prior to olfactory ablation, but in both cases, olfactory ablation resulted in complete loss of HD cell tuning (Supplementary Fig. 5), again indicating that, at least in this experimental paradigm, the vibrissal system was not being used to tune HD cells. These results show that blind mice use olfactory cues to anchor their HD system.

We next examined the effect that olfactory ablation had on HD cells in blind animals. To test this, used an alternative method to reliably identify HD cells in ADn regardless of whether or not they exhibited strong tuning preferences for head direction. Previous work^50^ noted that HD cells in ADn exhibit highly distinctive autocorrelograms. Similarly, we found that HD cells in ADn maintain their distinctive autocorrelograms in blind animals and following OSN ablation (Fig. 5f, Supplementary Fig. 5). Following Veijo and Peyrache^50^, we implemented a machine learning approach, using Extreme Gradient Boosting^51^ (XGB) to classify neurons as either HD or non-HD cells based on the shape of their autocorrelogram. First, we used our standard method to define cells as either HD cells (which rely on cells having highly selective preferred firing directions for a given head direction; see Methods) or non-HD cells, and trained the classifier using an equal number of HD and non-HD cells from WT_L_ mice. To assess model generalizability, here, the classifier was validated on data from blind controls (pooled rd1 and Gnat1/2^mut^ mice; Fig. 5g). Furthermore, the classifier characterized a similar percentage of cells in ADn in blind animals with intact OSNs and following OSN ablation (Fig. 5h; see Methods). We, therefore, used the classifier to define HD cells in ADn of blind mice following OSN ablation, allowing us to compare their response properties with HD cells in blind animals with intact OSNs. By using the same model to identify HD cells in blind controls and OSN ablated animals, we found that OSN ablation led to a significant decrease in vector length (Fig. 5i). These results indicate that removing olfaction from blind animals results in untuned HD cells in ADn.

### Olfaction can modulate HD cell tuning in sighted animals

**Fig. 6:**
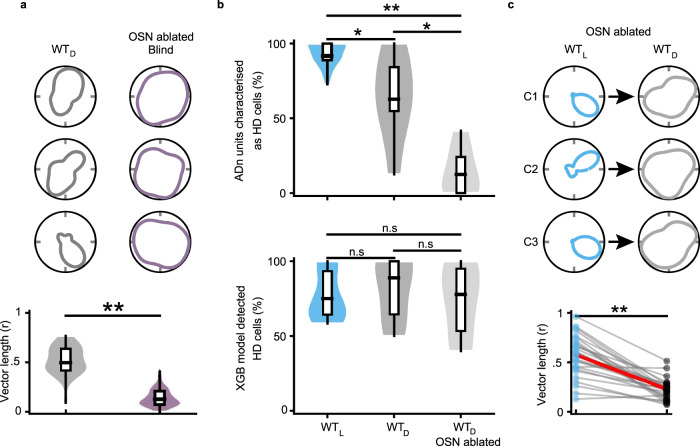
Olfaction modulates HD cell tuning in sighted mice placed in the dark. **a** top, Example polar plots from three simultaneously recorded HD cells in a sighted animal placed in the dark (WT_D_, left) and a blind rd1 mouse following olfactory sensory neuron ablation (right). **a** bottom, The vector length of tuning curves of HD cells belonging to blind animals following olfactory ablation is compared to that of sighted animals placed in the dark (36 HD cells from 6 animals for WT_D_; 166 HD cells from 9 animals for OSN ablated Blind). HD cells in both conditions were defined with an XGB model trained on blind control data. Statistical comparison was computed with the two-sided Mann–Whitney U Test, *P* < 10^−17^. **b** top, Violin/box plots are shown comparing the percent of recorded cells in ADn that passed the standard criterion for being designated as HD cells in WT_L_ (*n* = 9 animals), WT_D_ (*n* = 6 animals), and WT_D_: OSN ablated (*n* = 5 animals; see Methods). **b** bottom, Same as above except the percent of ADn units were defined as HD cells using the XGB classifier (see Methods). Statistical comparison was computed with the two-sided Mann–Whitney U Test with Bonferroni correction for multiple comparisons (*P < 0.05, **P < 0.01). **c** top, Example polar plots of three simultaneously recorded HD cells from a sighted animal following olfactory sensory neuron ablation, placed in either the light (left) or dark (right). **c** bottom, Vector length of tuning curves for HD cells from sighted animals placed in both light vs. dark environments following olfactory sensory neuron ablation (comparison of additional metrics is shown in Supplementary Fig. 6). *N* = 27 HD cells across 5 animals in both light and dark conditions. Statistical difference was calculated using the two-sided Wilcoxon Signed-Rank Test, *P* < 10^−5^. Source data are provided as a Source Data file.

We next examined whether olfaction could also impact HD cell tuning in sighted animals, as we noted that HD cell tuning was more robust in sighted animals in the dark (WT_D_) than in blind animals following OSN ablation (Fig. 6a). We thus wondered whether the weak but remnant tuning of HD cells in sighted animals in the absence of visual inputs could be arising from olfactory cues. To test this, we performed floor rotation experiments. Unlike for blind animals, HD cells in sighted animals in the dark did not exhibit significant PFD shift towards the direction of floor rotation (Supplementary Fig. 6a). However, upon ablating OSNs in sighted animals, the percentage of cells recorded in ADn that passed the criteria to be designated as HD cells (using our standard method) significantly decreased in WT_D_ (Fig. 6b), while the number of HD cells in ADn defined with an XGB model trained on blind data did not change (Fig. 6b), indicating that ablating olfaction significantly impaired the tuning of HD cells of WT animals in the dark. Ablating olfaction did not significantly affect the number of cells in ADn characterized as HD cells in WT animals in the light (Supplementary Fig. 6b), meaning that for these animals, turning the lights on and off toggled HD cell tuning from normal to completely untuned (Fig. 6c; Supplementary Fig. 6c). Thus, sighted animals can use both vision and olfaction to provide cue anchoring information to the HD system, though in the open-field environment we used for experiments, vision appears to be more effective at tuning the HD system. Only in the absence of both visual and olfactory cues does the HD system become completely untuned.

### Attractor dynamics in the absence of allothetic (external) sensory cues

**Fig. 7:**
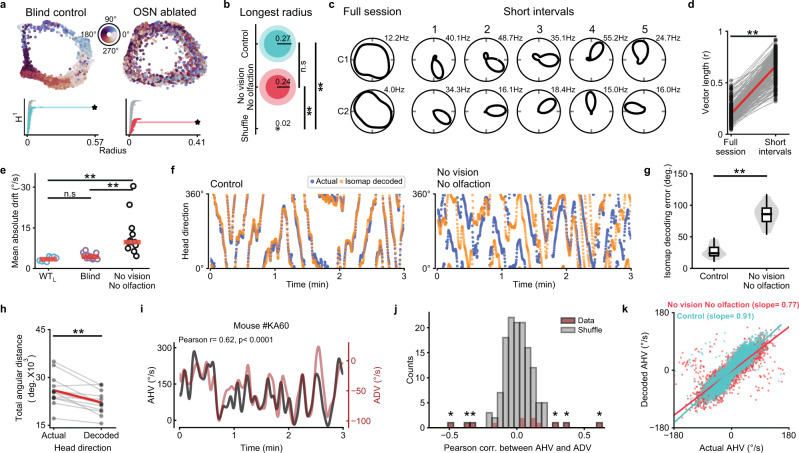
In the absence of vision and olfaction, the HD attractor in ADn remains intact but the ‘hill’ of activity drifts over time. **a** top, Isomap plots representing HD cell population activity over time, for a control blind rd1 mouse (left) and the same animal following olfactory sensory neuron ablation (right). The dots represent population activity at a given point in time, and when plotted over time form a 1-dimensional ring. Each dot is color-coded based on the animal’s actual head direction measured at that time point (additional plots for WT, rd1, and Gnat1/2^mut^ mice are shown in Supplementary Fig. 7a–c). **a** bottom, For the corresponding Isomap plots above, Betti 1 barcode plots are shown for actual and shuffled (gray) data (see Methods). **b** For control (blue; *n* = 13 animals (WT_L_ = 5, rd1 = 4, Gnat1/2^mut^ = 4)), ‘No Vision No Olfaction’ (red; *n* = 9 animals (WT_D_ = 2, rd1 = 4, Gnat1/2^mut^ = 3)) and shuffled (gray) Isomap data, the average length (solid color, inner circle) and standard deviation (opaque color, outer circle) of the longest (most persistent) radius are shown. Statistical difference computed with the two-sided Mann–Whitney U Test with Bonferroni correction for multiple comparisons (**P < 0.01). **c** Example polar plots for two simultaneously recorded HD cells in an rd1 mouse following OSN ablation, either calculated over the entire 10 min recording session (left) or over shorter timescales (right) with each successive epoch computed upon successive 360° head turns. **d** The mean vector length computed from HD cell tuning curves is compared when measured for the entire 10 min recording sessions or for the 360° head turn epochs across mice following loss of vision and olfaction. *n* = 209 HD cells from 14 animals (WT_D_ = 5, rd1 = 5, Gnat1/2^mut^ = 4). Statistical comparison with the two-sided Wilcoxon Signed-Rank Test, *P* < 10^−35^. **e** Average velocity of drift in the HD population compared in sighted mice in the light (WT_L_), blind mice, and in mice without both vision and olfaction. WT_L_ (*n* = 5 animals in 8 sessions); Blind (*n* = 8 animals in 13 sessions); No vision/olfaction (*n* = 9 animals in 12 sessions). Statistical comparison with the two-sided Mann–Whitney U Test with Bonferroni correction for multiple comparisons (**P < 0.01). **f** Comparison between an animal’s actual head direction vs. the head direction indicated by the population activity for blind mice before and after olfactory ablation. **g** Decoding error in control (*n* = 13 animals) and following loss of vision and olfaction (*n* = 9 animals). Statistical comparison with the two-sided Mann–Whitney U Test, *P* < 10^−5^. **h** Total angular distance covered for mice over the 10 min recording session compared to the total angular distanced covered on the Isomap manifold (*n* = 9 animals in 12 sessions). Statistical comparison with the two-sided Wilcoxon Signed-Rank Test, *P* = 0.0009. **i** Example blind mouse recording comparing angular head velocity (AHV) and angular drift velocity (ADV) following olfaction ablation. **j** Histogram showing the Pearson r and *p*-values computed for AHV versus ADV for all sessions where visual and olfactory inputs were blocked (as in panel **i**). Asterisk indicates sessions where *P* < 0.05 and |r values | > 99th percentile of shuffles (see Methods). **k** Scatterplot showing the relationship between actual and Isomap decoded AHV in Control (*n* = 13 animals) and No vision No olfaction (*n* = 9 animals). *P* = 0.025. Statistical comparison using the two-sided Mann–Whitney U Test. Source data are provided as a Source Data file.

We next tested how HD attractor dynamics were affected following the loss of cue anchoring visual and olfactory signals. We found that in the absence of both visual and olfactory cues, while HD cells became completely untuned, the activity of simultaneously recorded cells in ADn was well-described by a one-dimensional ring manifold generated with Isomap^52^ (Fig. 7a), similar to the HD network in blind control animals (Fig. 7a). A persistent homology topological analysis computed on the Isomap data^19^ was consistent with a one-dimensional ring architecture being preserved in the absence of vision, as well as in the absence of both vision and olfaction, even though in the absence of both vision and olfaction the population activity on the ring manifold representing the moment-to-moment head direction of the animal became decoupled from the actual head direction (Fig. 7a–c; Supplementary Fig. 7a–c; note that for the ‘No Vision and No Olfaction’ group, we pooled together blind animals following olfactory ablation and sighted animals in the dark (WT_D_) following olfaction ablation). For such a ring manifold to persist in the absence of visual and olfactory inputs, the relative spike timing of HD cells with respect to one another must be maintained as the animal moves around the environment, even though each cell no longer maintains a stable preferred firing direction over time. How could this arise? Previous work has shown that prior to eye-opening, while HD cell tuning is broad when measured over an extended period of time, if tuning curves are computed on short timescales then much more refined tuning curves can be measured^22^. Therefore, instead of computing tuning curves by averaging data across 10 min recording sessions as we have done until now, following the removal of both visual and olfactory inputs, we computed tuning curves each time an animal made an angular head rotation of 360° (which occurred on average every 25.4 ± 10.3 sec; see Methods). On these shorter intervals, we found that HD cells were highly tuned (Fig. 7c, d) and exhibited tuning curves similar to HD cells in control conditions (Supplementary Fig. 7d, e), but that their preferred firing directions continuously drifted over time, with all simultaneously recorded cells drifting in a coherent manner (Fig. 7c). Thus, in the absence of cue anchoring visual and olfactory signals, though HD cell tuning becomes unstable over long periods of time, the attractor network in ADn remains intact and HD cells exhibit sharp tuning curves over short time intervals.

We further examined the drift of the preferred firing direction of HD cells in the absence of both visual and olfactory inputs. To visualize this, we performed decoding using the Isomap analysis (Fig. 7f). We noticed that while the decoding became significantly impaired (Fig. 7g), the decoded head direction appeared to sometimes co-vary along with changes in actual head direction (Fig. 7f), though the total angular distance traveled by the animal was larger than the total decoded angular distance traveled (Fig. 7g). To test if a correlation existed between the animal’s head rotations and drift of preferred firing directions within the HD network, we compared angular head velocity signals (AHV; which is the vestibular signal provided to the head direction network) to angular drift velocity (ADV; which is a measure of the speed of the population drift of decoded head direction; see Methods). We found that in a subset of sessions, AHV and ADV were significantly correlated (Fig. 7i, j), suggesting that in at least some animals, the drift in preferred firing directions of HD cells was modulated by ongoing head rotations. Finally, as it has been previously reported in rodents before eye-opening that the broad tuning curves of their HD cells arise in part from ‘under-signaling’ of AHV signals, with the error between actual AHV and decoded AHV increasing as a function of actual AHV^22^, we tested if a similar phenomenon might underlie poor HD cell tuning in the absence of both vision and olfaction. To test this, we measured the error between AHV and decoded AHV (decoded using the Isomap analysis) and found that in the ‘No vision, No olfaction’ group, decoded AHV undershot the actual AHV, particularly for higher AHV values (Fig. 7k). Therefore, while the HD attractor network is intact in the absence of visual and olfactory inputs, either visual or olfactory landmarking cues are required to anchor the attractor network and enable stable HD cell tuning. Without such allothetic external sensory cues, the preferred firing direction of HD cells becomes unanchored and drifts, such that the HD network in ADn no longer encodes the animal’s actual head direction. For some animals, the drift of preferred firing directions appears to be coupled to ongoing angular head velocity signals, and at least part of the error in properly decoding head direction appears to arise from the under-signaling of angular head velocity signals.

## Discussion

Recording from head direction cells in ADn of both sighted and blind mice, we find stable and robust HD tuning in all animals. Remarkably, the HD system is flexible in which sensory system it can use for obtaining reliable anchoring to environmental landmarks: sighted animals predominantly use visual signals, whereas blind animals use olfactory signals. Additionally, there appears to be a critical period soon after eye-opening in which vision is required to fully refine and mature the HD system. Finally, in the absence of both visual and olfactory cues, the HD attractor network remains intact, but lacking any environmental anchors to lock onto, preferred firing directions of HD cells drift over time.

### External (allothetic) sensory cues are required for stabilizing the head direction system

The essential input to the HD system is thought to be vestibular—neurotoxic lesions of the vestibular nerve abolished HD cell tuning in ADn^23,25^. In contrast, though vision is known to play a strong role in landmarking the HD cell system^12^, since many studies have found relatively stable HD cell tuning following the removal of familiar visual cues or when animals were placed in the dark^14,26–28^, it has been argued that animals only require self-generated idiothetic sensory cues for stabilizing HD cell tuning^12,31,32^. However, a purely idiothetic mechanism for HD cell stability appears unlikely, since, with an exclusively internal path-integration system, each error and imperfection would be left uncorrected, likely leading over time to an unstable system. Our data is consistent with a model in which idiothetic (internal) and allothetic (external) sensory signals play complementary roles in stabilizing HD cell tuning: ablating olfaction in blind animals, and placing sighted animals in the dark following olfactory ablation both lead to total loss of HD cell tuning stability. This means that animals require externally generated, allothetic environmental cues processed via either visual or olfactory systems, or both together, in order to stabilize the preferred firing direction of HD cells in ADn. Our results also indicate that idiothetic cues alone (for instance arising from vestibular, proprioceptive, or motor efference copy systems) are insufficient to stabilize HD cell tuning in ADn, at least over long periods of time. Furthermore, our results suggest that previous studies describing stable HD cell tuning in the absence of visual inputs likely arose from either insufficient removal of all visual cues or from the presence of olfactory cues. Thus, vestibular and visual/olfactory signals appear to play complementary roles in stabilizing HD cell tuning in ADn.

Aside from visual and olfactory inputs, our results rule out a contribution of vibrissal signals in stabilizing HD cell tuning within our specific experimental environment, consistent with recent findings from the somatosensory cortex^38^. Nonetheless, one can imagine that in a different environmental context (e.g., where the walls or object contours provide useful spatial information) that the vibrissal system could possibly affect HD cell tuning. Next, since our experiments were done in the presence of white noise, we did not directly test possible auditory control of HD cell tuning. As such, future studies will be required to test for the possible use of auditory cues in stabilizing HD cell tuning in blind mice, though previous work in blindfolded rats suggests that auditory cues do not exert a strong effect on HD cell tuning^31^. However, it should be noted that a recent study found that some mice can echolocate^53^.

### Olfaction, HD cell tuning, and cognitive maps

Our results indicate that vision and olfaction can be used, either independently or in tandem, to stabilize HD cell tuning. Visual signals are believed to enter the HD system via inputs from the visual cortex to the post-subiculum and retrosplenial cortex^12^. With respect to olfactory inputs, the pathway into the HD system remains unclear, though direct projections from the olfactory bulb and piriform cortex to the entorhinal cortex^54,55^ could, in turn, reach ADn via post-subiculum, which is reciprocally connected to the entorhinal cortex and ADn^56^. Alternatively, olfactory signals could reach the HD system indirectly via the projection of the post-subiculum to the lateral mammillary bodies, which constitute the main subcortical input to the ADn^12^. Future studies examining these pathways in more detail in both sighted and blind mice will be required to develop a better understanding of the circuitry involved.

We find that, at least in our open-field environment, vision promotes the most refined HD cell tuning, with olfaction driving very robust but slightly less refined HD tuning in blind mice. Furthermore, blind mice appear more capable of using olfaction to stabilize HD cell tuning than sighted animals, which could relate to the finding that blind mice appear to have larger olfactory bulbs and perform better on some olfactory tests than sighted controls^57^. Overall, our results are consistent with a foraging study in rats that posited a sensory hierarchy in spatial navigation, with vision being at the top of the hierarchy, followed by olfaction^58^, though it is possible that such a hierarchy could change depending on the exact nature of the environment and which sensory system is most informative in a given condition.

Outside the HD cell system, a recent study has shown that spatial maps can be found in the piriform cortex during a spatial-olfactory learning task, with spatially encoding piriform cortex cells being linked to hippocampal theta^37^. Another recent study found that olfactory cues can modulate place cell responses in the hippocampus and aid in path integration^36^. Consistent with these findings, earlier studies in rats found intact place cells in blind animals^59^, as well as a contribution of olfactory inputs to place cell stability^60^. Therefore, seeing as olfactory inputs can enable robust and stable place cell and HD cell coding in the brain, olfaction needs to be considered an important sensory system for the generation of cognitive maps.

What olfactory cues do mice use to landmark their HD system? Previous work indicates that rodents can use a variety of odors, including self-generated odors, conspecific odors, and other non-animal odors, to guide spatial navigation^36,37,61^. In our experiments, for each mouse, its first open-field exposure began on a new floor, which had never been explored by another mouse, meaning the mice had to use cues intrinsic to the floor or that were self-generated.

How does a mouse use olfaction to guide spatial navigation? Odor cues that are located on the floor, which appear to be the cues the animals used in our experiments to guide spatial awareness, would likely smell the same when approached from every direction. Thus, the animal would likely need to consider more than a single odor cue in order to gain useful spatial information. In such a scenario, the animal would need to distinguish spatial location by the relative intensity of multiple distinct odor cues. Furthermore, as the intensity of the olfactory cue will change based on the animal’s distance from the cue, it would be advantageous if the mouse was actively monitoring multiple odor gradients during navigation. Is such a strategy feasible for mice? First, rodents appear capable of smelling in stereo^62^, which would greatly enhance olfactory spatial resolution and olfactory navigation capabilities. Second, there is evidence from experiments in rodents that olfactory coding can function in ‘snapshots’—i.e., discreet olfactory coding bouts linked to each sniff^63^—with a single sniff being sufficient for fine odor discrimination^64^. Third, rodents can identify a specific odor within a mixture of odors^64,65^. As such, it appears that the rodent olfactory system functions in a manner that would enable it to effectively serve spatial navigation needs.

### Vision around the time of eye-opening is required for refining HD cell tuning

Hubel and Wiesel discovered a critical period during development for ocular dominance plasticity in the visual cortex^66^, and similar critical periods have been shown to exist for many other sensory systems and behaviors^67^. Here, we provide evidence of a critical period in the refinement and maturation of the HD system in ADn that depends on visual inputs in the period shortly after eye-opening—the evidence being that rd1 mice, who have attenuated vision upon eye-opening before going blind around P30^40,47^, have more refined HD cell tuning as adults than congenitally blind Gnat1/2^mut^ mice. Both rd1 and Gnat1/2^mut^ mice go blind due to problems with retinal photoreceptors. rd1 mice go blind as a result of photoreceptor degeneration caused by a mutation in phosphodiesterase in rod photoreceptors (due to a mutation of the *Pde6B* gene), which initially leads to rod death followed by cone death, and this is a commonly used mouse model of retinitis pigmentosa^39^. Gnat1/2^mut^ mice are blind as a result of mutations in both rod and cone forms of the alpha subunit of the G-protein transducin (due to mutations in both *Gnat1* and *Gnat2* genes), resulting in nonfunctioning rod and cone photoreceptors (see Methods). As the mutated genes in both mouse lines are predominantly expressed in photoreceptors, it is likely that the difference in HD cell tuning in blind adult Gnat1/2^mut^ vs. rd1 mice is a direct result of the timing of the onset of vision loss (congenital vs. ~P30). We thus propose that vision around the time of eye-opening allows the HD cell system to stabilize (i.e., upon eye-opening, vision enables HD cells to exhibit heightened stability in their preferred direction tuning^29,30^), and this stability results in refinement and maturation of the HD network, such that though both rd1 and Gnat1/2^mut^ are equally blind as adults, rd1 mice have more refined HD cell tuning. These findings are consistent with multisensory studies in the superior colliculus, which showed the importance of vision during development for enabling the generation of normal auditory space maps^43,44,68^.

### Attractor dynamics in the presence and absence of anchoring external sensory inputs

The HD network is often modeled as a continuous ring attractor^11,18–20^. Evidence in favor of an attractor network is plentiful, from the finding that the relative difference in preferred directions between a given pair of HD cells is maintained across different environments and during visual cue rotation experiments^21^, to the finding that HD cells that fire coherently during spatial navigation continue to do so during sleep^11^, to the finding that before eye-opening—when HD cell tuning curves are broad—pairs of cells exhibit similar coherence during spatial navigation as is seen in the adult HD system^22,29,30^. Furthermore, a recent manifold analysis has validated a one-dimensional ring as a robust description of the rodent HD network^19^. Our results in blind mice are consistent with those of a continuous ring attractor. Performing a manifold analysis revealed a one-dimensional ring attractor similar to that found in normally sighted animals. Additionally, upon ablating OSNs in blind animals, while this completely removed stability of HD cell preferred direction, attractor dynamics and the ring architecture remained intact.

We find that—for normally sighted and blind animals—the removal of both visual and olfactory inputs causes the ‘hill of activity’ within HD attractor network to drift independently from the animal’s true head direction (i.e. the preferred direction of all simultaneously recorded cells drifts coherently). For some animals, there was a significant correlation between angular head velocity and angular drift velocity of PFDs. Furthermore, we found that in the absence of vision and olfaction, the decoded angular velocity (AHV) under-signaled the true AHV.

A similar drift in HD cell preferred firing direction appears to have been described in a previous study recording from HD cells in the lateral dorsal nucleus in rats placed in the dark, though those animals were placed in a radial maze, making it difficult to draw exact parallels to our results^33^. In a more recent study in mice placed in the dark, it was reported that ~40% of HD cells became unstable in the dark, with one example HD cell recording being shown where the instability resulted in the preferred firing direction exhibiting a drift similar to what we describe^34^. However, a follow-up paper from the same lab showed no effect of dark exposure on HD cell stability and instead found that optogenetic silencing of the nucleus prepositus hypoglossi (NHP)—which relays vestibular signals to the HD system—was required in tandem with dark exposure to cause the preferred direction of a subset of HD cells recorded in ADn to drift over time^14^. This latter study also found that preferred firing direction drift was sometimes correlated with the animal’s head turns^14^. Finally, at least some portion of the drift in HD preferred firing directions that we find appears to be related to the effect described for HD cells prior to eye-opening, where the broadness of tuning curves resulted in part from an under-signaling of angular head velocity^22^. Nonetheless, future work is required to better understand the circuit and synaptic basis whereby different sensory inputs enable tuning within the HD attractor network to stabilize, and how perturbations to different sensory modalities can alter the input to the HD network and result in drift.

### Relating head direction cell recordings in blind rodents to spatial cognition in visually impaired humans

While vision is often considered the most important sensory system for guiding spatial cognition in humans, numerous studies have shown that visually impaired individuals maintain robust spatial cognition^4,5,69,70^. Unlike in blind mice—where olfaction appears to be the most important sensory system for guiding spatial awareness following vision loss—in visually impaired humans, the auditory system is generally thought to be a more important contributor to vision-independent spatial awareness, with blind humans appearing to exhibit enhanced sound localization compared to sighted subjects^71^ and performing echolocation via self-produced sounds^72–74^. However, there is emerging evidence that humans can also effectively use olfaction to inform spatial cognition^75–77^, even being able to navigate using stereo olfaction^78^, meaning that olfaction could be used following vision loss to aid in spatial awareness and navigation^79^. Relatedly, visually impaired humans appear to have enhanced olfactory abilities compared to sighted individuals^80–83^, which could further help visually impaired individuals use olfaction to guide spatial awareness and navigation. Next, while there is active debate surrounding the extent to which visual experience in humans is required for developing normal spatial cognitive abilities^4,6^, some studies have indicated that late-blind individuals perform better on certain spatial cognition tasks than congenitally blind individuals^84–87^, reminiscent of our findings regarding HD cell tuning being slightly more refined in rd1 mice compared to congenitally blind Gnat1/2^mut^ mice. Thus, our findings related to HD cells in sighted and blind mice are likely highly relevant for understanding the neural underpinnings of spatial cognition in humans following vision loss.

## Methods

### Animals

All procedures were performed in accordance with the Canadian Council on Animal Care and approved by the Montreal Neurological Institute’s Animal Care Committee. Three strains of mice were used in this study: wild-type mice (C57Bl/6; Charles River strain code 027), rd1 mice (also known as C3H; The Jackson Laboratory #000661), and Gnat1/2^mut^ mice (Gnat2^cpfl3^ Gnat1^irdr^/Boc mice; The Jackson Laboratory #033163). All mice were adults (2–4 months old; P75-125) weighing between 20 and 32 g. Mice of both sexes were included. Mice were maintained on a standard 12 h light, 12 h dark cycle, in ventilated and humidity-controlled racks, at standard room temperature.

### Surgery and implantation

Mice were anesthetized with a cocktail containing fentanyl (0.05 mg/kg), medetomidine (0.5 mg/kg), and midazolam (5 mg/kg)^88^, and a craniotomy was performed above ADn for probe implantation. A conductive wire was inserted into the cerebellum to serve as a reference. After attaching the reference wire, a 4-shank silicon probe (Neuronexus Inc. Ann Arbor, MI; 200 µm inter-shank spacing) with 8 recording sites on each shank (probe model Buz32) was lowered into the brain towards ADn based on the following stereotactic coordinates: antero-posterior (AP) −0.4 mm; medio-lateral (ML) −0.76 mm; dorso-ventral (DV) 2.16 mm. The base of a movable drive holding the silicon probe was then fastened to the skull using dental acrylic cement and a light-cure adhesive (Kerr OpitBond Universal Unidose) to allow for stable recordings during awake open-field sessions.

### Electrophysiological recordings

After recovery (5–7 days postsurgery), during sleep, the probe was advanced daily in steps of <300 µm in the home cage until HD units were detected in ADn. Prior to open-field recording sessions, screenings were done during sleep on the homepage. Preliminary detection of putative HD cells was based on inspection of autocorrelograms of recorded units, as previously described^50^. Once putative HD units were detected, experiments were conducted by placing animals in an open-field dark cylindrical arena (60 cm in diameter) with a single white visual cue placed on the wall above the animal’s reach. Open-field recordings lasted for 10 min per session. The neurophysiological signals were acquired at 20 kHz using an Intan RHD2000 Recording System (16-bit, analog plexin). The raw neuronal signal was high-pass filtered and processed with an automated spike sorting algorithm to extract single units (Kilosort2^89^). Isolated units were manually curated in Klusters^90^ based on autocorrelograms and spike waveforms. For a given animal, after encountering cells with the recording electrode, the electrode was advanced ~100 µm per day, and thus cells from a given animal that were recorded on different days were considered to be different cells, meaning that a single animal could contribute multiple ‘sessions’ with unique cells to a given experiment—the only exception being the recordings across days following OSN ablation shown in Fig. 5d, where cells were recorded over a 2-day period, and the sessions were merged to form a single continuous recording before spike sorting to ensure that the same units were maintained for further analysis.

### Position tracking

For position tracking, an infrared-based (850 nm IR) camera recording system equipped with 8 cameras recording at 120 FPS was mounted above the recording arena to capture the movements of the animal (Optitrack Flex 13). Four reflective infrared markers were attached to the animal’s head-stage for tracking (6.4 mm Optitrack M3 Markers). After recording, Motive motion capture software (Optitrack) was used to extract both the direction and position of the head relative to the environment in three dimensions. It should be noted that for dark experiments, we found that sighted mice could see the visual cue in ‘the dark’ when we used the 850 nm IR illumination that came with the Optitrack cameras (i.e., HD cells followed the visual cue in ‘the dark’ when it was moved to a new location (Supplementary Fig. 1)). This is consistent with findings from a recent paper about red light perception in rats^91^. As such, for dark experiments, we installed a custom-made array of 940 nm infrared LEDs (Adafruit, Product # 388) that we used for illumination and marker detection. Importantly, under 940 nm IR illumination HD cells did not exhibit visual cue-controlled changes in their preferred firing direction.

### Standard HD cell classification

To identify HD cells, as mentioned above, we first used autocorrelograms to localize the electrode in ADn and identify putative HD cells located in ADn^50^. Subsequently, for all cells recorded in ADn, tuning curves were constructed by aligning spike times with the closest head direction point in the horizontal plane. Spikes were then counted for bins of 6°. To correct for angular sampling bias, spike rates were normalized by the time spent in each angular bin, as previously described^11^. The resulting tuning curves representing the normalized firing rate of each cell as a function of head direction were then smoothed with a Gaussian kernel (s.d. = 3). Units that passed a Rayleigh test for significant non-uniform circular distribution (*p* < 0.0001), had a z-test score > 50, and a peak firing rate > 1 Hz, were classified as HD cells.

### Extreme gradient boosting (XGB) model for HD cell classification

Owing to the loss of stable HD cell tuning in blind animals following olfactory sensory neuron ablation, cells recorded in ADn in this condition were classified as HD/non-HD cells using an XGB model^50,51^, implemented in Python. In control animals (sighted animals in the light (WT_L_)) we used the standard HD cell classification method (see above) to define cells in ADn as either HD or non-HD cells. Next, we generated autocorrelograms from these HD and non-HD cells using 2 ms bins and a window of 400 ms. To capture potential variability in HD autocorrelogram shapes over the course of each session, autocorrelograms were generated from the 1st and 2nd half of each recording session before stacking them together. Since autocorrelograms are symmetrical, for further processing, we only used the halves corresponding to the positive lag. The following pre-processing steps were carried out prior to model training. First, we ensured that the classes (HD/non-HD) were equally represented in the training set. Second, each half of the stacked autocorrelograms was smoothed with a Gaussian kernel (s.d. = 1.5). Next, using default parameters, the XGB model was trained on all pre-defined (i.e., standard-method-labeled HD and non-HD cells) autocorrelograms, and then the performance was evaluated on the data from blind mice (pooled between rd1 and Gnat1/2^mut^ mice). The model performance on the test data had an accuracy of 90% and an F1 score of 90.5% in correctly classifying cells recorded in ADn of blind animals (Fig. 5g). Next, the trained model was used to classify ADn cells recorded in blind animals following OSN ablation as either HD or non-HD cells based on their autocorrelograms (Fig. 5h, i). In addition, in order to use the XGB model to define HD cells in WT_D_ following OSN ablation (Fig. 6b), we trained the model on HD cells recorded from blind animals to ensure no data leakage between the testing and training set.

### Visual cue rotation

The extent to which the single white cue card on the arena wall controlled the preferred firing direction (PFD) of HD cells was tested by rotating the visual cue by either 90° or 180°. Following a standard session where the baseline PFDs for all simultaneously recorded HD cells was established, the animal was removed from the arena and disoriented, as previously described^10^. The animal was then reintroduced to the arena after the visual cue was rotated. For all visual cue rotation experiments, the floor of the arena was thoroughly cleaned with 70% ethanol to eliminate olfactory cues on the floor before the animal was reintroduced. The visual cue control measure (gain) shown in Fig. 1g was computed as follows:

1 $${{{{{{\mathrm{Gain}}}}}}}=\,\left|\triangle {{{{{{\mathrm{PFD}}}}}}}\right|/\,\left|\triangle {{{{{{\mathrm{cue}}}}}}}\right|$$

where, $\triangle$PFD is defined as the change in the preferred firing direction (in degrees) for each HD cell following reintroduction into the arena, and $\triangle {{{{{{\mathrm{cue}}}}}}\; {{{{{\mathrm{is}}}}}}}$ defined as the extent (either 90° or 180°) that the visual cue was rotated.

### Floor rotation

To assess floor-controlled changes in PFDs of HD cells, a protocol similar to the visual cue rotation described above was adopted, except where the floor of the arena was rotated. For these experiments, to avoid possible interference of odor markings across different animals, a brand new floor was used for each mouse. At the same time, to give mice the opportunity to use olfactory odors on the floor, the floor of the arena was not cleaned between exposures. The extent to which floor rotations influenced the PFD of HD cells across animals was computed using Eq. (1). For cue/floor rotation sessions, 10,000 shuffles were generated using re-exposure sessions where animals were re-exposed to the same environment in the absence of any manipulation. To generate the gain values expected by chance, the gain was computed after randomly applying the experimental cue/floor rotation angles to the re-exposure data.

### Olfactory sensory neuron (OSN) ablation

To eliminate (or at least greatly reduce) the sense of smell, OSNs were chemically ablated based on established methods^48,92^. In brief, mice were anesthetized with isoflurane after which a blunted 33-gauge needle was used to administer 20 μl of ZnSO4 (10 % in sterile H_2_O) in both nostrils. Previous work has shown that this leads to the rapid death of OSNs^48,92^. After intranasally administering the chemical, mice were inverted to drain the excess fluid from the nasal cavity. Behavioral and electrophysiological recordings were carried out at least 24 h following OSN ablation.

### 2-Chamber olfactory test

To test the efficacy of OSN ablation, in some treated animals, we tested their sense of smell using a 2-chamber odor test. Animals were first habituated to the chambers for 5 min each day over a period of 3 days, with no odors added to the chambers. After habituation, testing began by placing two filter papers infused with 10 µl of 3-methyl-1-butanethiol (aversive odor^49^) in a randomly selected chamber, and two filter papers infused with 10 µl of distilled water (neutral odor) in the other chamber. All mice were placed in the neutral chamber at the start of the test and were monitored for 10 min using an overhead camera to record their baseline occupancies in both chambers. Post-OSN ablation, the test was repeated, and compared to the baseline. The chamber preference (CP) score in Fig. 5 was computed as follows:

2 $${{{{{{\mathrm{CP}}}}}}}=\frac{{t}_{{{{{{{\mathrm{neut}}}}}}}}-{t}_{{{{{{{\mathrm{avers}}}}}}}}}{{t}_{{{{{{{\mathrm{neut}}}}}}}}+{t}_{{{{{{{\mathrm{avers}}}}}}}}}$$

where *t*_neut_ and *t*_avers_ refers to the time spent in the neutral and aversive chamber, respectively. Chamber preference scores of −1 and 1 represent a strong preference for the aversive and neutral sides, respectively, where the animal spent the entire time in one chamber.

### Bayesian decoding

To predict the HD of the animal, given the spiking activity of HD cells within a given time window, a Bayesian decoding algorithm^41^ was used to compute the posterior probability distribution using the formula:

3 $$P\left({{{dir}}}/{{{spk}}}\right)=\frac{P\left({{{spk}}} {/}{{{dir}}}\right) * P\left({{{dir}}}\right)}{P\left({{{spk}}}\right)}$$

where *dir* represents the set of possible angular head directions, and *spk* represents the spike counts of simultaneously recorded HD cells within a 200 ms time window in this instance. Bayesian decoding was used to predict the HD of animals in the second half of a recording session based on priors generated from tuning curves in the first half. The mean absolute decoding error was computed as the absolute difference between the decoded angle and the actual angle over the decoded time window.

### Isomap projection

Isomap analysis was performed on sessions with at least 10 HD cells based on a threshold previously used^50^. Similar to Viejo and Peyrache^50^, spike counts were binned (200 ms) and square-root transformed to normalize for variance in spike rates. The normalized rates from all simultaneously recorded units in ADn served as inputs to the Isomap algorithm^52^, implemented in Python^93^. The number of neighbors was set to 50. The resulting output of the algorithm formed the basis of the low dimensional ring manifolds shown in Fig. 7a and Supplementary Fig. 7a–c. The color code of the population vector on the ring manifold was mapped onto the corresponding actual HD for each corresponding time bin. Isomap decoded angular head velocity was generated by taking the angular difference between two consecutive points after computing the element-wise arc tangent of the point cloud. For Isomap shuffles, each cell’s binned spike counts were randomly shifted in time to generate a shuffled matrix before applying the Isomap algorithm (Fig. 7a, Supplementary Fig. 7a–c).

### Drift analysis

Following the loss of vision and olfaction, the population drift was computed using the Isomap ring manifold to provide a fine temporal resolution. The drift was computed with the following steps. First, the actual HD was down-sampled to match the time bin (200 ms) used for computing the Isomap ring manifold. Following this, the formula below was used to compute the angular difference (Ang_diff_) between the actual and Isomap decoded HD.

4 $${{{{{{{\mathrm{Ang}}}}}}}}_{{{{{{{\mathrm{diff}}}}}}}}={\arctan }2\left({{{{{\rm{sin }}}}}}\left({{{{{{{\mathrm{HD}}}}}}}}_{{{{{{{\mathrm{actual}}}}}}}}-{{{{{{{\mathrm{HD}}}}}}}}_{{{{{{{\mathrm{iso}}}}}}}}\right),\; {{{{{\rm{cos }}}}}}\left({{{{{{{\mathrm{HD}}}}}}}}_{{{{{{{\mathrm{actual}}}}}}}}-{{{{{{{\mathrm{HD}}}}}}}}_{{{{{{{\mathrm{iso}}}}}}}}\right)\right)$$

where HD_actual_ and HD_iso_ refers to the actual and Isomap decoded HD, respectively. Next, the angular difference over time was unwrapped and smoothed with a Gaussian kernel (s.d. = 2), from which drift was computed as the absolute average rate of change (Fig. 7e). The shuffles shown in Fig. 7j were computed by correlating the AHV of each animal to the ADV of all other animals to generate a null distribution of AHV vs ADV correlations.

### Quantification of ADn units

To ensure that cells counted on recording shanks were recorded in ADn, the following was implemented: first, autocorrelograms were generated from all cells on shanks that picked up at least 1 HD cell. Second, a Fourier transform was applied to the autocorrelograms to identify and exclude theta (4–8 Hz) modulated cells. Although some studies have reported the presence of theta modulation in surrounding anterior thalamic structures^94,95^, a recent study found that cells in ADn are not theta modulated^50^. Hence the total counts of ADn cells were derived from all non-theta modulated units. Finally, the proportion of ADn units characterized as HD cells was derived from the total number of units classified as HD cells (see Methods: HD classification) divided by the total number of cells recorded in ADn. In Figs. 2 and 3, the difference between proportions was tested using a 2-sample Z-test for proportions as previously described^22^.

### Analysis of head direction cell

Mean firing rate: The temporal average of spike counts. Peak firing rate: The normalized maximum spike counts in a second. Mean vector length: The circular spread of spikes, with 0 and 1 representing strong uniform and non-uniform circular spread, respectively. In Fig. 7d, for each cell, the mean vector length for the short intervals was computed by averaging the vector length values across all 360° head turns in a session. To control for biases introduced by relative differences in spike counts, for each epoch, a corresponding number of spikes was randomly selected from the full session before computing the vector length. Tuning width: The full width at a half max of a cell’s tuning curve. Mutual information: An estimate of the directional information relating the firing rate of a cell to a given direction. The formula^96^ is given as follows:

5 $$I=\sum {P}_{j}\frac{{\lambda }_{j}}{\lambda }{{\log }}_{2}\left(\frac{{\lambda }_{j}}{\lambda }\right)$$

where *I* refers to the information content in bits/spike*, P*_*j*_ is the probability of occupying bin *j, λ*_*j*_ is the mean firing rate in bin *j*, and *λ* is the in-session mean firing rate of the cell. Preferred firing direction (PFD): Similar to Lozano et al.^97^, PFD was defined as the angular bin with the highest normalized spike counts, whereas the mean PFD refers to the circular mean of angles. In Fig. 1e, stability was assessed based on the circular correlations of mean PFDs of exposure 1 and 2 across all recorded animals. Circular statistics were computed using the astropy module (v5.1) in Python.

### Intraclass correlation (ICC)

We used ICC to assess the degree of correlation due to clustering of HD cells within an animal/session vs. across animals/sessions^98^. In this instance, each session from an animal within a given strain (e.g., WT_L_, rd1, etc.) is considered as a class where the population ICC metric evaluates the ratio of the between-class variance to the total strain variance with respect to measured metrics based on the formulation below:

6 $${{{{{{\mathrm{ICC}}}}}}}=\,\frac{{\sigma }_{b}^{2}}{{\sigma }_{b}^{2}+{\sigma }_{e}^{2}}$$

where ${\sigma }_{b}^{2}$ denotes the between-class variance and ${\sigma }_{e}^{2}$ denotes the within-class variance.

Here, for each strain, it was used to determine if cells from individual animals/sessions were less homogenous compared to the group. ICC scores normally fall between 0 and 1. ICC = 0 suggests the absence of correlation due to clustering, whereas an ICC of 1 suggests a strong correlation due to clustering, which implies that there is greater homogeneity within cells from individual animals and greater heterogeneity between cells across animals; hence, cells should not be treated as independent samples. Based on published guidelines^99^, ICC scores below 0.5 are considered acceptable for treating cells across animals as independent samples (related to Supplementary Figs. 2a and 3d). ICC analyses were performed in RStudio (2022.02.1 Build 461) using the ‘ICC’ package^100^.

### Statistical analyses

The statistical test used for every specific comparison is directly stated in the text or figure legend. For hybrid violin/box plots, the violin plot shows the density distribution of all data points, whereas the box-plot shows the median, 25/75% distribution, and 5/95% distribution. All violin plots were scaled to have the same width of 0.6 using matplotlib’s width parameter. For statistical comparisons, *p < 0.05, **p < 0.01.

### Reporting summary

Further information on research design is available in the Nature Research Reporting Summary linked to this article.

## Supplementary information

Supplementary Information

Reporting Summary

## Data Availability

Source data are provided in the supplementary material. All data used for analyses can be found here: https://github.com/kadjitaa/Research/tree/main/HeadDirectionCells/Asumbisa_et_al_2022. Source data are provided with this paper.

## Source data

Source Data

## Code availability

Custom code can be found here: https://github.com/kadjitaa/Research/tree/main/HeadDirectionCells/Asumbisa_et_al_2022.
