# Supplementary material for: Flexible cue anchoring strategies enable stable head direction coding in both sighted and blind animals
Source: Nat Commun. 2022 Sep 19;13:5483. doi: 10.1038/s41467-022-33204-0 (PMC9485117; doi:10.1038/s41467-022-33204-0)
Supplement: Supplementary file 1 — Supplementary Information [file 41467_2022_33204_MOESM1_ESM.pdf]

Supporting Information:

Supplementary Figures 1-7

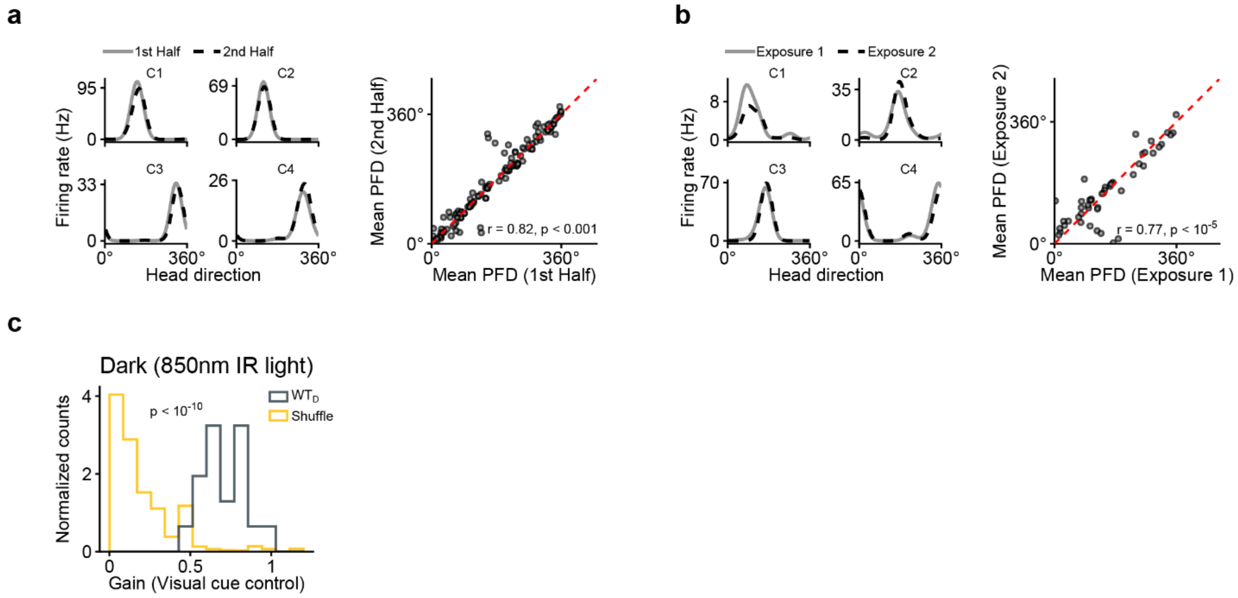

Supplementary Figure 1. a, left, The spike rate vs. head direction of 4 simultaneously recorded HD cells (C1-C4) in a sighted mouse during the first and last 5 minutes of a 10-minute recording session. a, right, For all HD cells (128 HD cells across 9 animals), the mean PFD is compared between the first and last 5 minutes. The pre-post tuning similarity was tested with a circular correlation, and the resulting r value is shown. b, The same as in a, except comparing HD cell tuning across successive 10-minute exposures to the same open field arena (48 HD cells across 4 animals). c, Histogram showing the extent of control (gain) that visual cue rotation exerted on the PFD of HD cells in sighted mice in the dark under 850nm IR light (see Methods). Gain distributions for WT_D mice were compared to a shuffled distribution. n = 18 HD cells across 2 animals, two-sided Mann Whitney U Test, $P < 10^{-10}$. Source data are provided as a Source Data file.

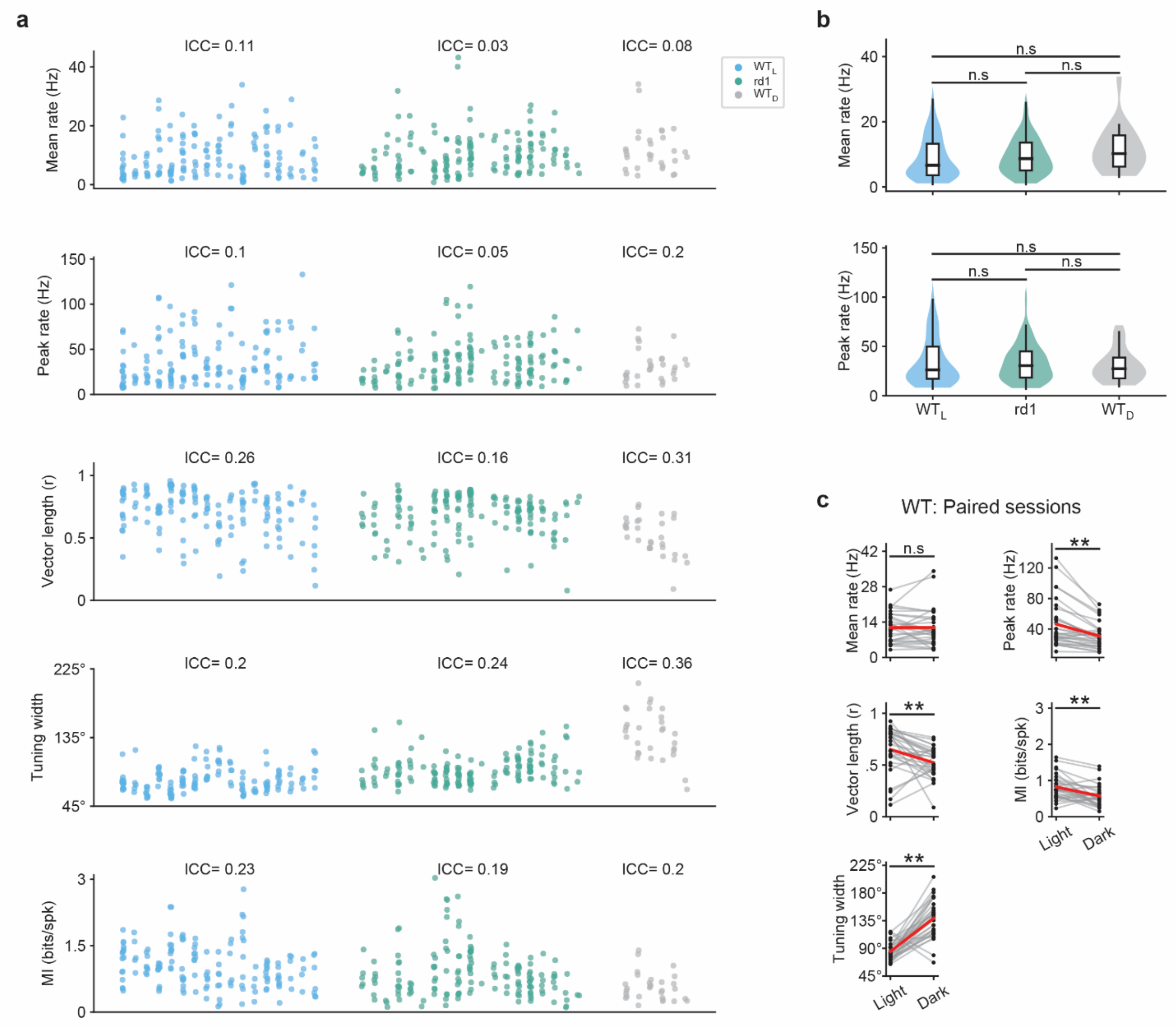

Supplementary Figure 2. a, Intra-class-correlation (ICC; see Methods) scores showing that cells are less homogeneous in variance within an animal compared to the group variance, across all measured metrics, for WT$_L$, rd1 and WT$_D$ (see Methods). WT$_L$ (n = 128 HD cells from 9 animals over 17 sessions), rd1 (n = 151 HD cells from 13 animals over 19 sessions) and WT$_D$ mice (n = 30 HD cells from 6 animals). b, Mean and peak rates between WT$_L$ (n = 128 cells from 9 animals), rd1(n = 151 HD cells from 13 animals) and WT$_D$ mice (n = 30 HD cells from 6 animals). Statistical difference was tested with the two-sided Mann Whitney U Test with Bonferroni correction: Mean rate (WT$_L$ vs rd1, P = 0.29; WT$_L$ vs WT$_D$, P = 0.11; rd1 vs WT$_D$, P = 0.5); Peak rate (WT$_L$ vs rd1, P = 1; WT$_L$ vs WT$_D$, P = 1; rd1 vs WT$_D$, P = 1). c, Comparison of metrics for WT$_L$ vs WT$_D$ paired sessions (n = 30 HD cells from 6 animals). Statistical difference was tested with the two-sided Wilcoxon Signed-Rank Test: P = 0.78 (Mean rate); P < 10^{-4} (Peak rate); P = 0.007 (Vector length); P = 0.0005 (MI); P < 10^{-4} (Tuning width). Source data are provided as a Source Data file.

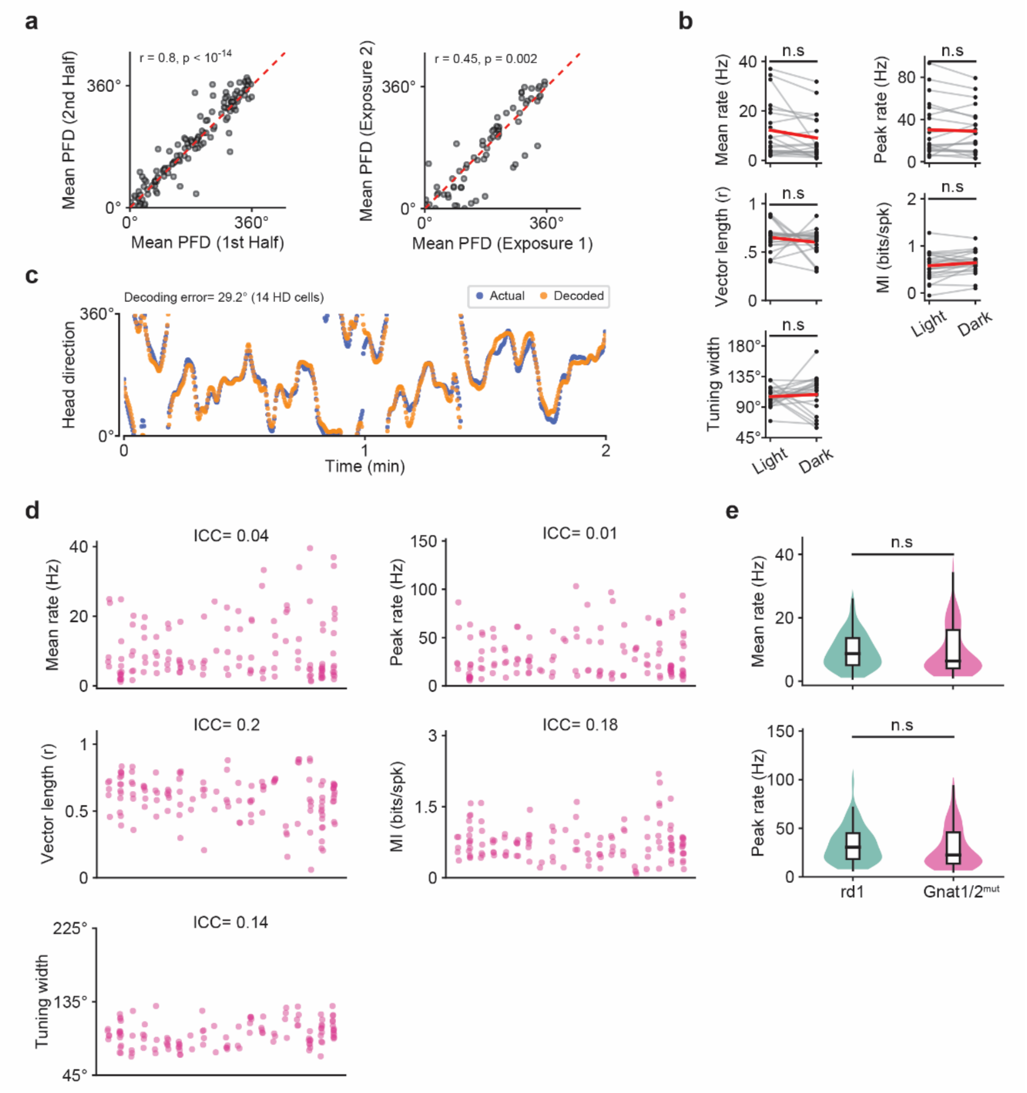

Supplementary Figure 3. a, left, For all HD cells from Gnat1/2^{mut} animals (128 HD cells across 8 animals), the mean PFD is compared between the first and last 5 minutes. The pre-post tuning similarity was tested with a circular correlation, and the resulting r value is shown. a, right, The same as in a, left, except comparing HD cell tuning across successive 10-minute exposures to the same open field arena (70 HD cells across 5 animals). B, Several metrics characterizing HD cells in Gnat1/2^{mut} mice are compared during light vs. dark exposure (21 HD cells across 3 animals). Statistical differences were calculated using the two-sided Wilcoxon Signed-Rank Test, n.s = not statistically different. c, Analysis comparing the actual head direction of a Gnat1/2^{mut} mouse over time (blue) to the head direction predicted by a Bayesian decoder (orange; see Methods). d, Intra-class-correlation (ICC; see Methods) scores showing that cells are less homogeneous in variance within an animal compared to the group variance, for all measured metrics (n = 128 HD cells from 8 animals over 20 sessions). e, Mean and peak spike rates compared between rd1 (n = 151 HD cells from 13 animals) and Gnat1/2^{mut} mice (n = 128 HD cells from 8 animals). Statistical difference was tested with the two-sided Mann Whitney U Test. Source data are provided as a Source Data file.

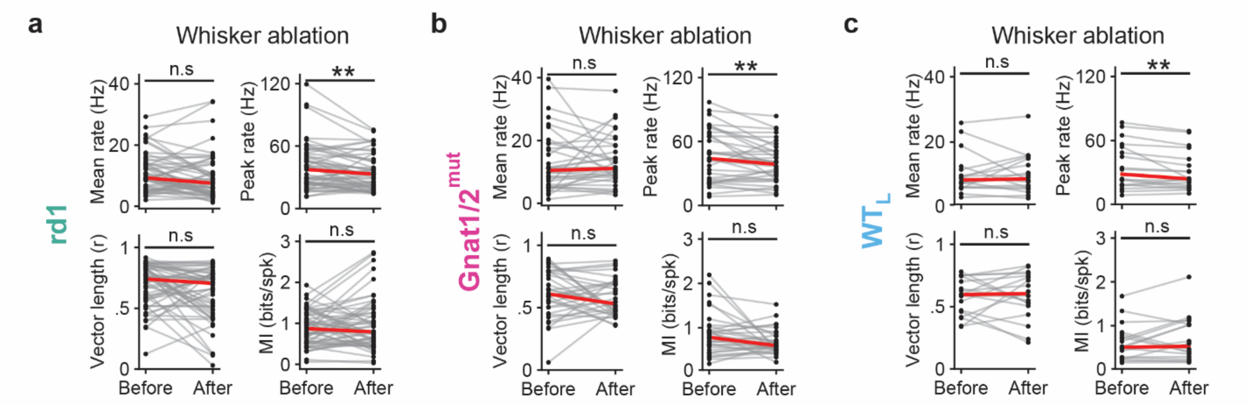

Supplementary Figure 4. Plotting several metrics for HD cells before and after whisker shaving in rd1 (a; n = 55 HD cells from 7 animals), Gnat1/2^{mut} (b; n = 34 HD cells from 4 animals) and WT_L (c; n = 18 HD cells from 3 animals) mice. Statistical difference was tested with the two-sided Wilcoxon Signed-Rank Test. rd1 (Mean rate, P = 0.07 ; Peak rate, P = 0.005; Vector length, P = 0.06; MI, P = 0.43 ; Gnat1/2^{mut} (Mean rate , P = 0.98; Peak rate, P = 0.009; Vector length, P = 0.34; MI, P = 0.09; WT_L (Mean rate, P = 0.7; Peak rate, P = 0.007; Vector length, P = 1; MI, P = 0.32. Source data are provided as a Source Data file.

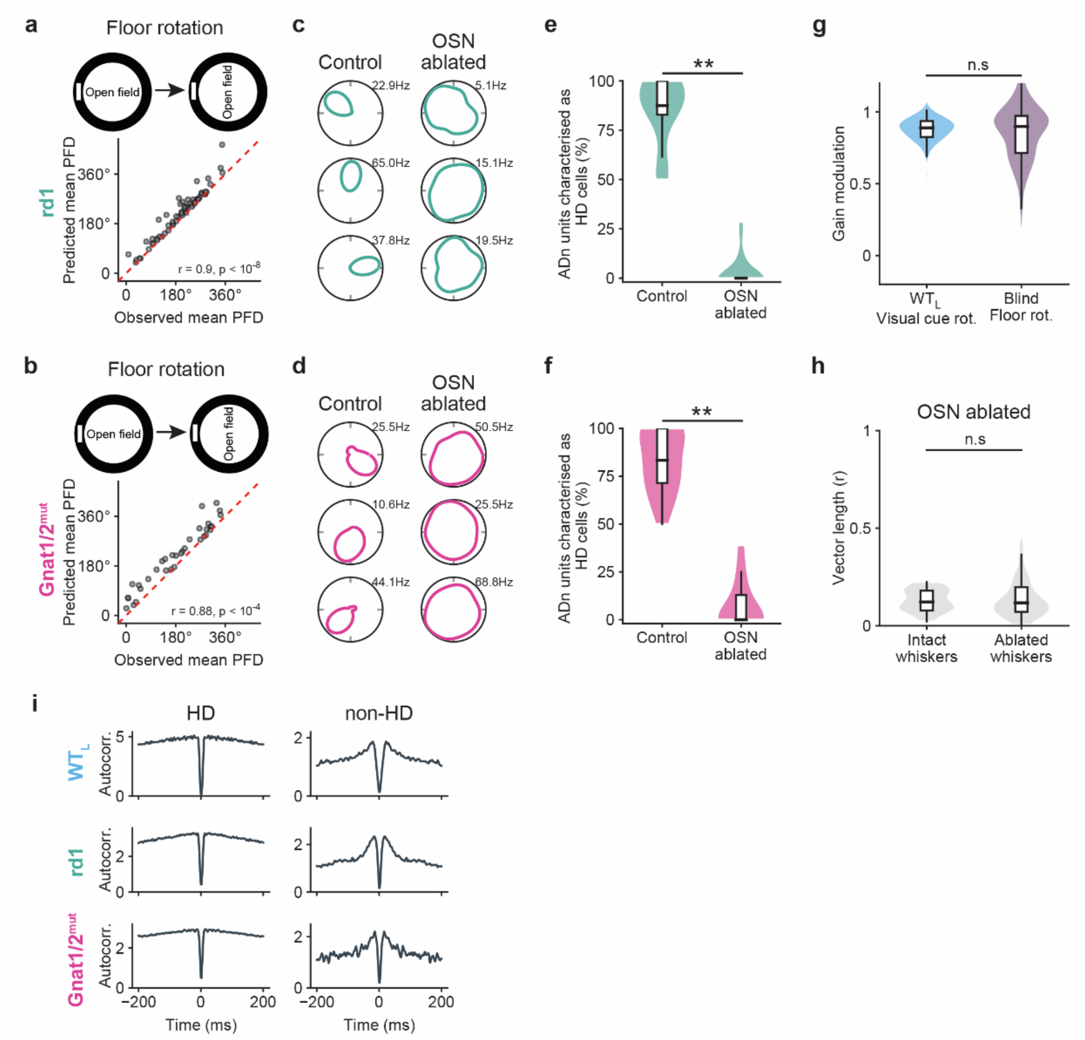

Supplementary Figure 5. a, b, Related to Figure 5a, showing the observed shift in the mean PFDs plotted against the expected shift in mean PFDs for rd1 (n = 60 HD cells from 5 animals) and Gnat1/2^{mut} (n = 34 HD cells from 4 animals) mice respectively. The r values listed were computed with a circular correlation. c, d, Showing example HD cells before and after OSN ablation in rd1 and Gnat1/2^{mut} mice, respectively. e, f, The % of units characterized as HD cells before and after OSN ablation is shown separately for rd1 (e; n = 5 animals) and Gnat1/2^{mut} (f; n = 4 animals) mice. g, The extent to which visual cue/floor rotation exerted control on the PFD of HD cells (visual cue for sighted animals in the light (WT_L; n = 93 HD cells from 6 animals) and floor rotation for blind animals (pooled rd1 and Gnat1/2^{mut} mice; n = 94 HD cells from 9 animals)), measured as the gain between the extent of HD cell PFD rotation compared to the extent that the visual cue/floor was rotated. Statistical difference was tested with the two-sided Mann Whitney U Test. h, Comparison of the vector length of HD cells recorded in blind mice following olfaction ablation, with and without whiskers. Intact whiskers = 54 HD cells; Ablated whiskers = 125 HD cells. Statistical difference was tested with the two-sided Mann Whitney U Test. i, Example autocorrelograms for HD and non-HD cells across mouse strains, used for XGB model training. ** P < 0.01. Source data are provided as a Source Data file.

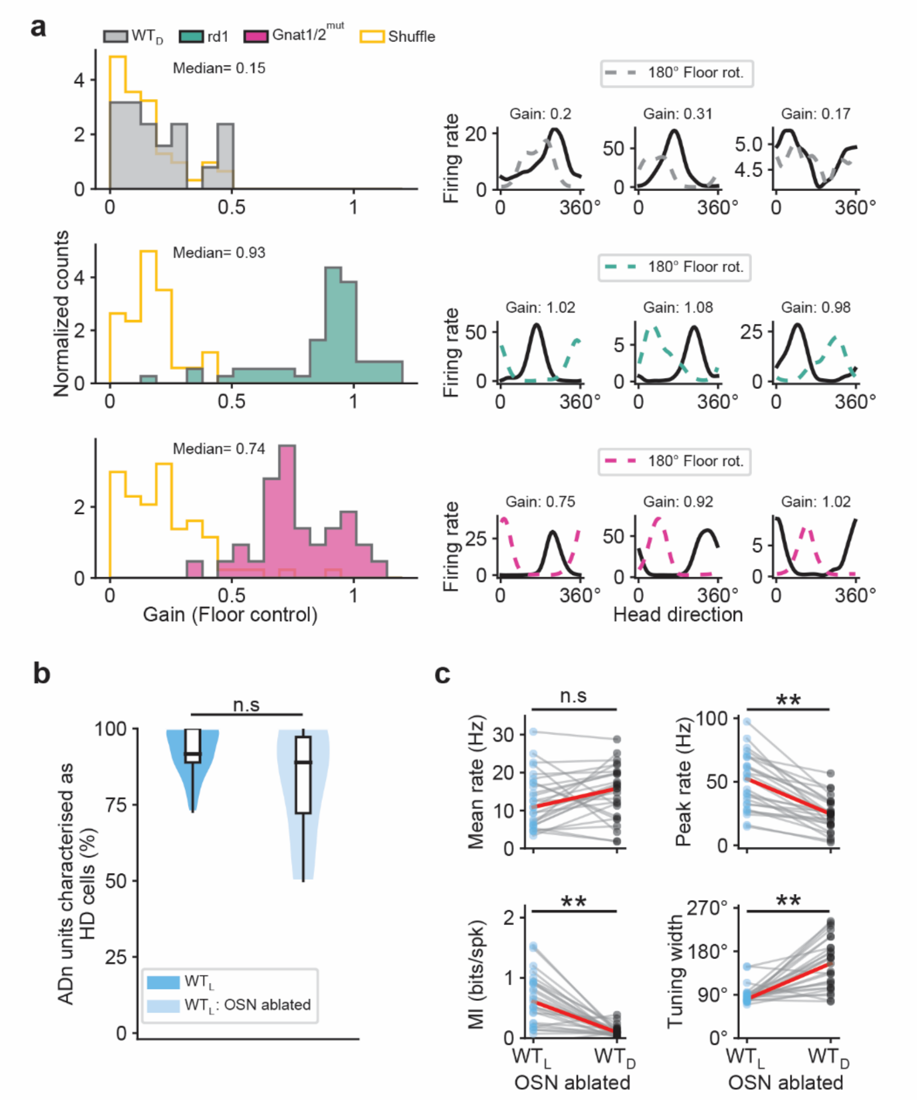

Supplementary Figure 6. a, left, Histogram showing the extent of control the floor rotation (gain) exerted on the PFD of HD cells for different groups of mice: sighted animals in the dark (WT_D; n = 20 HD cells from 4 animals; P = 0.11), rd1 (n = 60 HD cells from 5 animals; $P < 10^{-38}$) and Gnat1/2^{mut} (n = 34 HD cells from 4 animals; $P < 10^{-20}$) mice. Statistical difference comparing gain to shuffles was tested with the two-sided Mann Whitney U Test. a, right, For each of the different groups of mice listed on the left (see color legend), the spike rate vs. head direction of 3 example HD cells in control condition (solid black line) and following a 180° floor rotation (dotted line). b, Comparison of the % of ADn units characterized as HD cells in sighted animals placed in light before (WT_L = 9 animals) and after olfactory sensory neuron ablation (WT_L: OSN ablated = 5 animals). Statistical difference was tested using the two-sided Mann Whitney U Test, P = 0.23. c, Several metrics of HD responses from sighted animals placed in both light vs. dark environments following olfactory sensory neuron ablation (n = 27 HD cells across 5 animals). Statistical differences were calculated using the two-sided Wilcoxon Signed-Rank Test: Mean rate, P = 0.06; Peak rate, $P < 10^{-5}$; Tuning width, $P < 10^{-4}$; MI, $P < 10^{-4}$. Source data are provided as a Source Data file.

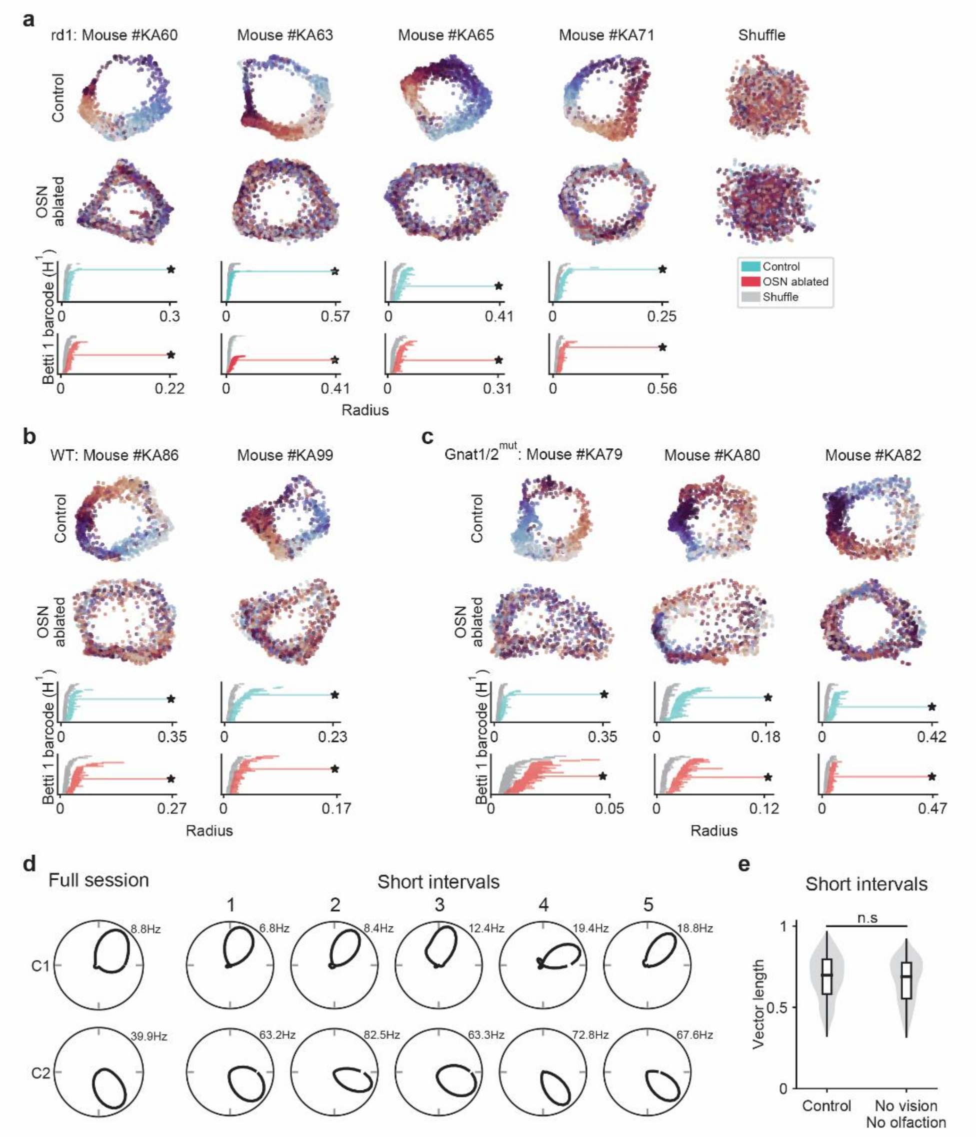

Supplementary Figure 7. Example Isomap plots outlining the 1-dimensional ring manifold of the HD cell network in control and olfaction ablation sessions in (a) rd1 mice, (b) sighted wildtype animals (control (in the light), vs. in the dark following OSN ablation), and (c) Gnat1/2^{mut} mice. Corresponding Betti 1 barcode plots are shown below the manifolds, with the actual data color coded, and the shuffled data (see Methods) in grey. Example shuffled Isomap plots are shown in (a) for mouse #KA71. d, Example polar plots for 2 simultaneously recorded HD cells in a sighted mouse in the light, either calculated over the entire 10-minute recording session (left) or over shorter timescales (right) with each successive epoch computed upon successive 360° head turns (for comparison to Figure 7c). e, Comparison between the responses of HD cells computed on short timescales (each time the animal makes a 360° head rotation) compared between control animals (combined sighted wildtypes in the light and blind mice; n = 407 HD cells from 30 animals) and animals with No vision and No olfaction (n = 209 HD cells across 14 animals). Statistical difference was tested with the two-sided Mann Whitney U Test, P = 0.1. Source data are provided as a Source Data file.
