# Supplementary material for: Flexible cue anchoring strategies enable stable head direction coding in both sighted and blind animals
Source: Nat Commun. 2022 Sep 19;13:5483. doi: 10.1038/s41467-022-33204-0 (PMC9485117; doi:10.1038/s41467-022-33204-0)
Supplement: Supplementary file 2 — Reporting Summary [file 41467_2022_33204_MOESM2_ESM.pdf]

nature portfolio

Corresponding author(s): Stuart Trenholm

Last updated by author(s): Aug 25, 2022

Reporting Summary

Nature Portfolio wishes to improve the reproducibility of the work that we publish. This form provides structure for consistency and transparency in reporting. For further information on Nature Portfolio policies, see our Editorial Policies and the Editorial Policy Checklist.

Statistics

For all statistical analyses, confirm that the following items are present in the figure legend, table legend, main text, or Methods section.

n/a	Confirmed	
☐	☒	The exact sample size (*n*) for each experimental group/condition, given as a discrete number and unit of measurement
☐	☒	A statement on whether measurements were taken from distinct samples or whether the same sample was measured repeatedly
☐	☒	The statistical test(s) used AND whether they are one- or two-sided<br>*Only common tests should be described solely by name; describe more complex techniques in the Methods section.*
☒	☐	A description of all covariates tested
☐	☒	A description of any assumptions or corrections, such as tests of normality and adjustment for multiple comparisons
☐	☒	A full description of the statistical parameters including central tendency (e.g. means) or other basic estimates (e.g. regression coefficient) AND variation (e.g. standard deviation) or associated estimates of uncertainty (e.g. confidence intervals)
☐	☒	For null hypothesis testing, the test statistic (e.g. *F*, *t*, *r*) with confidence intervals, effect sizes, degrees of freedom and *P* value noted<br>*Give P values as exact values whenever suitable.*
☒	☐	For Bayesian analysis, information on the choice of priors and Markov chain Monte Carlo settings
☒	☐	For hierarchical and complex designs, identification of the appropriate level for tests and full reporting of outcomes
☐	☒	Estimates of effect sizes (e.g. Cohen's *d*, Pearson's *r*), indicating how they were calculated

Our web collection on statistics for biologists contains articles on many of the points above.

Software and code

Policy information about availability of computer code

Data collection	Commercial softwares: Motive (OptiTrack) version 2.2.1, Intan RHD2000 Recording System (16-bit, analog plexin)
Data analysis	Commercial software: Matlab (R2019a)<br>Open source: Neurosuite, klusters, RStudio, (python (3.5), anaconda (2.1.1), spyder (5.1.5), scipy (1.3.1), numpy (1.20.3), pandas (1.3.4), astropy (v5.1), Ripser (0.6.3), neuroseries (0.1), scikit-learn (1.0.2), custom code (https://github.com/kadjitaa/Research/tree/main/HeadDirectionCells/Asumbisa_et_al_2022/)

For manuscripts utilizing custom algorithms or software that are central to the research but not yet described in published literature, software must be made available to editors and reviewers. We strongly encourage code deposition in a community repository (e.g. GitHub). See the Nature Portfolio guidelines for submitting code & software for further information.

Data

Policy information about availability of data

All manuscripts must include a data availability statement. This statement should provide the following information, where applicable:

- Accession codes, unique identifiers, or web links for publicly available datasets
- A description of any restrictions on data availability
- For clinical datasets or third party data, please ensure that the statement adheres to our policy

Source data are provided in the supplementary material. All data used for analyses can be found here: https://github.com/kadjitaa/Research/tree/main/HeadDirectionCells/Asumbisa___et___al___2022

Field-specific reporting

Please select the one below that is the best fit for your research. If you are not sure, read the appropriate sections before making your selection.

☒ Life sciences ☐ Behavioural & social sciences ☐ Ecological, evolutionary & environmental sciences

For a reference copy of the document with all sections, see nature.com/documents/nr-reporting-summary-flat.pdf

Life sciences study design

All studies must disclose on these points even when the disclosure is negative.

Sample size	We chose the number of animals based on numbers reported in similar studies (Butler et al.,2017; Peyrache et., 2015) examining head direction cells in mice.
Data exclusions	Cells that did not pass the HD cell criteria (see Methods) were excluded.
Replication	Animal and cell counts were provided in all figures as well as identification of some animals to facilitate replication.
Randomization	For the chamber preference test, the aversive and neutral odor sides were pseudo-randomly assigned for each animal.
Blinding	Different strains of mice were visibly different from one another, so no blinding was used.

Reporting for specific materials, systems and methods

We require information from authors about some types of materials, experimental systems and methods used in many studies. Here, indicate whether each material, system or method listed is relevant to your study. If you are not sure if a list item applies to your research, read the appropriate section before selecting a response.

Materials & experimental systems

n/a	Involved in the study	
☒	☐	Antibodies
☒	☐	Eukaryotic cell lines
☒	☐	Palaeontology and archaeology
☐	☒	Animals and other organisms
☒	☐	Human research participants
☒	☐	Clinical data
☒	☐	Dual use research of concern

Methods

n/a	Involved in the study	
☒	☐	ChIP-seq
☒	☐	Flow cytometry
☒	☐	MRI-based neuroimaging

Animals and other organisms

Policy information about studies involving animals; ARRIVE guidelines recommended for reporting animal research

Laboratory animals	The following strains were used: C57Bl/6 (Charles River strain code 027); rd1 (Jackson Laboratory #000661); Gnat1/2mut (Gnat2cpfl3 Gnat1irdr/Boc mice; Jackson Laboratory #033163). For each strain, both male and female mice aged 2-4 months, and weighing between 20 - 32grams were used. Animals were maintained on 12hr light/dark cycle in humidity/temperature controlled racks
Wild animals	None
Field-collected samples	None
Ethics oversight	All procedures were performed in accordance with the Canadian Council on Animal Care and approved by the Montreal Neurological Institute's Animal Care Committee.

Note that full information on the approval of the study protocol must also be provided in the manuscript.
